# Landscape Heterogeneity and Environmental Dynamics Improve Predictions of Establishment Success of Colonising Small Founding Populations

**DOI:** 10.1111/eva.70027

**Published:** 2024-10-21

**Authors:** Arman N. Pili, Nathan H. Schumaker, Morelia Camacho‐Cervantes, Reid Tingley, David G. Chapple

**Affiliations:** ^1^ School of Biological Sciences, Faculty of Science Monash University Clayton 3800 Victoria Australia; ^2^ Macroecology, Institute of Biochemistry and Biology University of Potsdam Potsdam 14469 Brandenburg Germany; ^3^ US Environmental Protection Agency Pacific Ecological Systems Division Corvallis Oregon USA; ^4^ Instituto de Ciencias del Mar Y Limnología Universidad Nacional Autonoma de Mexico Mexico City Mexico

**Keywords:** Allee effect, colonisation, dynamic environmental conditions, establishment, landscape spatial heterogeneity, phenology, propagule pressure

## Abstract

In long‐distance dispersal events, colonising species typically begin with a small number of founding individuals. A growing body of research suggests that establishment success of small founding populations can be determined by the context of the colonisation event and the new environment. Here, we illuminate the importance of these sources of context dependence. Using a spatially explicit, temporally dynamic, mechanistic, individual‐based simulator of a model amphibian species, the cane toad (*Rhinella marina*), we simulated colonisation scenarios to investigate how (1) the number of founding individuals, (2) the number of dispersal events, (3) landscape's spatial composition and configuration of habitats (‘spatially heterogeneous landscapes’) and (4) the timing of arrival with regards to dynamic environmental conditions (‘dynamic environmental conditions’) influence the establishment success of small founding populations. We analysed the dynamic effects of these predictors on establishment success using running‐window logistic regression models. We showed establishment success increases with the number of founding individuals, whereas the number of dispersal events had a weak effect. At ≥ 20 founding individuals, propagule size swamps the effects of other factors, to whereby establishment success is near‐certain (≥ 90%). But below this level, confidence in establishment success dramatically decreases as number of founding individuals decreases. At low numbers of founding individuals, the prominent predictors are landscape spatial heterogeneity and dynamic environmental conditions. For instance, compared to the annual mean, founding populations with ≤ 5 individuals have up to 18% higher establishment success when they arrive in ‘packed’ landscapes with relatively limited and clustered essential habitats and right before the breeding season. Accounting for landscape spatial heterogeneity and dynamic environmental conditions is integral in understanding and predicting population establishment and species colonisation. This additional complexity is necessary for advancing biogeographical theory and its application, such as in guiding species reintroduction efforts and invasive alien species management.

## 1 Introduction

Species colonisation is arguably the most significant eco‐evolutionary process shaping global biodiversity and maintaining ecosystem function (Brown and Lomolino 1998; Davis and Thompson 2000; Stigall 2019; Wilson and MacArthur 1967). Colonisation is initiated by one to countless individuals—that is, "propagules"—dispersing to novel areas beyond their geographical ranges. Upon arrival, founding propagules face biotic and environmental filters to establish a self‐sustaining population. The colonisation process repeats when individuals of the established population spread by diffusing or dispersing to and establishing anew in neighbouring or distant novel areas, expanding their range in the process (Blackburn et al. 2011; Brown and Lomolino 1998; Hoffmann and Courchamp 2016; Jeschke, Keesing, and Ostfeld 2013; Wilson et al. 2009). At any stage along the dispersal–establishment cycle, colonising species can cause profound eco‐evolutionary consequences, from disrupting trophic structures (Wainright et al. 2021), undermining co‐occurring ‘species’ populations, pushing co‐occurring species to extinction (Blackburn et al. 2004; MacArthur and Wilson 2001; Wilson and MacArthur 1967), reshaping biotic community composition (biotic homogenisation; Capinha et al. 2015; Olden et al. 2004) and to altering ecosystem function (Castro‐Diez et al. 2019; Vilà and Hulme 2017). Under natural rates of species colonisation (Gaston et al. 2003), these eco‐evolutionary changes transpire over evolutionary timescales (MacArthur and Wilson 2001; Wilson and MacArthur 1967). However, at the turn of the Anthropocene, human‐mediated species dispersal dramatically increased species colonisation rates (Gaston et al. 2003; Gleditsch, Behm, and Helmus 2023; Seebens et al. 2018, 2017), abruptly bringing about eco‐evolutionary changes that toppled communities and ecosystems out of balance (Pysek et al. 2020; Vila et al. 2011; Vilà and Hulme 2017). Given its utmost significance to global biodiversity and ecosystem function, understanding the factors, processes and mechanisms that allow species to colonise new environments has been a central theme of theoretical and applied biogeographical research (Brown and Lomolino 1998; Drake et al. 1989; Simberloff 2010; Williamson et al. 1986).

The most decisive step in the species colonisation process is the transition from arrival to establishment. Under the lens of human‐mediated species colonisation (or more precisely, biological invasions; Blackburn 2004), establishment is principally determined by propagule pressure (i.e., the composite measure of the number of dispersal events [i.e., propagule number] and the number of individuals dispersing [i.e., propagule size]; Hayes and Barry 2007; Lockwood, Cassey, and Blackburn 2005). In a review by Cassey et al. (2018), experimental and observational studies across taxonomic groups and spatial scales showed that, although establishment is never certain, establishment probability increases with increasing propagule size of founding individuals. This consistency in pattern has led many to hypothesise propagule pressure as the *null model* of alien species invasions (Cassey et al. 2018; Cassey, Prowse, and Blackburn 2014; Colautti, Grigorovich, and MacIsaac 2006; Stringham and Lockwood 2021), and by extension, species colonisation. Nonetheless, this null model is arguably a deceptively simple premise whose interpretation and use warrant caution (Chapple, Simmonds, and Wong 2012).

The most crucial caveat of Cassey et al.'s (2018) proposed *null model* (i.e., propagule pressure) of alien species invasions is that establishment is conditional to the environment being suitable for species (Cassey et al. 2018; Chapple, Simmonds, and Wong 2012). Environmental conditions must first be conducive to maintaining vital life history processes and self‐sustaining a population (Devictor et al. 2010; Soberon 2007). In this sense, propagule pressure is fundamentally equally important, if not second, to environmental suitability in determining establishment success (Blackburn et al. 2011; Forsyth et al. 2004; Hayes and Barry 2007; Rago, While, and Uller 2012; Richardson et al. 2000; Roura‐Pascual et al. 2011). Secondly, even under suitable environments, propagule pressure weakly explains the establishment probability of populations with small numbers of founding individuals. Examining the raw empirical data collected in the Cassey et al. (2018) meta‐analysis, the establishment probability of populations founded by few (< 20) individuals ranged from 0% to 100%, resulting in disconcertingly wide error margins of their model's predictions at low propagule pressure. And this low predictability of small founding population's establishment success is taxing to our overall understanding of the species colonisation process, because small founding populations and infrequent dispersal events exemplify most natural and human‐mediated species colonisation events (Deredec and Courchamp 2007; Pili et al. 2023; Wilson et al. 2009).

The importance of the environment to species’ establishment probability is evinced in countless macroecological studies (Blackburn 2004; Blackburn and McGill 2019). However, at finer spatial and temporal scales, this relationship is often disparate or context‐dependent—that is, the magnitude or sign of relationships varies under different spatial, temporal, environmental or observational circumstances (Catford et al. 2022; Pickett and Cadenasso 1995). Unresolved sources of context dependence can be taxing in making generalisable insights and generating predictable and transferable relationships between ecological processes and the factors driving them (Buchadas et al. 2017; Catford et al. 2022; Cuddington et al. 2013; DeAngelis and Yurek 2016; Elliott‐Graves 2015; Pickett and Cadenasso 1995). This is especially pressing for applied disciplines, such as invasion science and reintroduction biology, which demand robust and defensible predictions and risk assessments of future invasions, reintroduction success probabilities and management efficacy (Kolar and Lodge 2002; Leung et al. 2012). Notably, only by understanding the mechanisms that allow a founding population to establish (or go extinct) can we identify the underlying sources of context dependence and, in turn, account for them in designing, interpreting and communicating research (Catford et al. 2022; Cuddington et al. 2013).

Two putatively pivotal fine‐scale environmental factors in species’ establishment probability, often overlooked if not dismissed as context‐dependent, are (1) landscape spatial heterogeneity and (2) dynamic environmental conditions. Landscape spatial heterogeneity—that is, the composition and configuration of habitats within the landscape, mediates key life history processes (O'Reilly‐Nugent et al. 2016; Pickett and Cadenasso 1995). For instance, in sexually reproducing mobile species, founding individuals would have to navigate through disparate habitats across a landscape to find a suitable breeding area with a suitable mate. In theory, breeding can be challenging at low population densities, typifying founding populations, as undercrowding limits the availability of suitable mates (i.e., Allee effect; Courchamp, Clutton‐Brock, and Grenfell 1999). Nonetheless, empirical studies have attributed the establishment of small founding populations to chance—that is, demographic and environmental stochasticity (Duncan et al. 2014; Melbourne and Hastings 2009). Meanwhile, individuals execute key life history processes (i.e., reproduction) according to the state of their dynamic environment (e.g., temperature and moisture levels), strategically to maximise their fitness (Calatayud, Stoops, and Durrant 2018; Frederiksen et al. 2004; Joly 2019; Segrestin et al. 2018). This leads to the emergence of species phenologies—that is, periodical life history phenomena, such as cyclical breeding seasons (Forrest and Miller‐Rushing 2010). Albeit conceptually pivotal, there remains an unsettling knowledge gap on how spatially heterogeneous landscapes and temporally dynamic environmental conditions mediate the establishment of founding populations and, by extension, species colonisation.

Here, we simulated species' colonisation scenarios to understand how propagule pressure, landscape spatial heterogeneity and dynamic environmental conditions (with regards to the timing of introduction) interplay in the establishment of small founding populations. To do this, we used *virToad*—a spatially explicit, temporally dynamic, mechanistic, individual‐based life history simulator of a model amphibian species, the cane toad (*Rhinella marina*), developed by Pili et al. (2022). In analysing the effect of dynamic environmental conditions, we developed a novel framework where simulation outputs are analysed using a running‐window approach. We asked the following:

1. Can a simple model that only accounts for propagule pressure reliably predict establishment success? [Hypothesis 1] As with the many studies on propagule pressure and establishment probability (Cassey et al. 2018), we predict establishment probability and confidence of predictions to increase with increasing propagule pressure. And extending this, we hypothesise that a simple model that only accounts for propagule pressure will produce highly uncertain predictions of establishment probability of small founding populations.
2. Would the establishment probability of a founding population vary in different landscapes? [Hypothesis 2] We predict that establishment success would vary in landscapes with different compositions and configurations of habitats. In the light of positive density dependence, wherein low population densities would limit the availability of suitable mates (Courchamp, Clutton‐Brock, and Grenfell 1999), we specifically predict that establishment probability will be higher in ‘packed’ landscapes—that is, landscapes with limited and clustered configuration of essential habitats for movement, survival and reproduction (O'Reilly‐Nugent et al. 2016). A packed landscape would result in the concentration of founding individuals in these essential habitats (relatively higher population densities), and thus, founding individuals would have a higher chance of finding suitable mates.
3. Would the establishment probability of a founding population vary with the timing of introduction? [Hypothesis 3] Considering species phenology, wherein critical life history processes, such as dispersal and reproduction, have annual cycles in many species (Forrest and Miller‐Rushing 2010), we predict that establishment probability would vary with the timing of introduction. We predict that founding populations introduced right before or during the breeding season would have a higher establishment probability.

Our study investigates two putatively crucial but widely overlooked sources of context dependence in species colonisation: how establishment success is mediated by landscape spatial heterogeneity and dynamic environmental conditions. We discuss herein how our findings pave the way for making generalisable insights and robust, defensible and transferable predictions of establishment success and, by extension, species colonisation. In the end, we discuss the benefits of accounting for these sources of context dependence in applied biogeography, such as managing invasive alien species and reintroducing species.

## 2 Methods

### 2.1 *virToad*—A Life‐History Simulator of the Cane Toad

We used the *virToad* computer simulator developed by Pili et al. (2022). *virToad* is a spatially explicit, temporally dynamic, mechanistic, individual‐based life history simulator of the cane toad. *virToad* is used for understanding how landscape structure and environmental conditions affect the cane toad's life history (i.e., individual life history traits, processes and behaviours), biotic and abiotic interactions and emergent local‐to landscape‐scale spatiotemporal population dynamics (i.e., density, distribution and spatial segregation). *virToad* can also be used to explore the effectiveness of cane toad management responses.

#### 2.1.1 Entites and State Variables

The key entities of *virToad* relevant in this study are the simulated cane toads. The simulated cane toads are modelled based on real‐world cane toads in Australia. Simulated cane toads are primarily classified based on their sex (male or female), the population of origin (long‐established, intermediate or invasion front), life phase and stage (aquatic phase: egg or tadpole; terrestrial phase: metamorph, juvenile or adult), hydric balance (hydrated, dehydrated, extremely dehydrated or desiccated) and behavioural activity (inactive, breeding, rehydrating or foraging).

#### 2.1.2 Spatial and Temporal Scales

*virToad* has a daily time step and is run in a simulation landscape made up of a two‐dimensional grid of hexagonal cells, with the distance between hexagon centres being 10 m. Each hexagon cell describes the habitat features on its location within the landscape.

#### 2.1.3 Input Data

The input data required to run a *virToad* simulation are a land cover map depicting the spatial arrangement of habitat types (water bodies, human habitation, grassland, woodland and road) and a table containing daily and cumulative rainfall records.

### 2.2 Design Concepts

The *basic principles* underpinning *virToad*’s design are the dependence of simulated toads on water and the implications of kin selection on individual survival and reproduction. Toads can *sense* their internal hydric balance, the landscape’s wetness, their surrounding habitats and the presence of other toads. Toads *adapt* their behaviour in light of environmental constraints and social interactions (and management actions), with the *objective* of maximising their fitness. Specifically, they maximise their fitness by foraging in high‐resource habitats, maintaining their hydric balance, optimising mating chances and optimising the survival of their offspring. Notably, many of the toad's life history processes (sex, behavioural decisions, dispersal movement, mate selection and mortality) are *stochastic*. Altogether, the individual toad's behavioural responses to environmental cues and constraints collectively lead to the *emergence* of spatiotemporal population dynamics. We further optimised *virToad'*s design to run cane toad colonisation scenarios (see Appendix S1).

### 2.3 Process Overview

1. *Life history*, *growth and development—*As in the real world, simulated cane toads have a semi‐aquatic amphibian life cycle. Toads start as eggs, hatch into tadpoles in 3 days (Hearnden 1991), metamorphose at 34–55 days (Crossland et al. 2011; Hearnden 1991) and reach adulthood and sexual maturity in an average of 243 days (Cohen 1995). Toads engage in social interactions during the aquatic phase when tadpoles cannibalise and chemically suppress eggs (mechanisms of kin selection; Crossland et al. 2011; Hearnden 1991), and during adulthood, when breeding toads search for spawning sites with no tadpoles and with calling adult male toads, and when they select a suitable mate (Clarke, Shine, and Phillips 2019; Hearnden 1991; McCann et al. 2020). Toads can perceive environmental moisture and distant foraging habitats, rehydration sites and breeding areas (Brodie et al. 2020; Carpenter and Gillingham 1987; Hearnden 1991; Jørgensen 1991; Yasumiba, Alford, and Schwarzkopf 2015). With the goal of maximising fitness, cane toads modify their behaviour in light of social interactions and environmental cues and constraints.
2. *Water Ecophysiology—*Terrestrial cane toads lose body weight daily due to water loss; they are dehydrated when they lose 10% of their body weight and they will be desiccated and die once they lose 40% (Jørgensen 1991; Schwarzkopf and Alford 1996; Seebacher and Alford 2002). To avoid desiccation, dehydrated toads will attempt to rehydrate. During the wet season or rainy days, toads can rehydrate from their immediate environment. In contrast, during the dry season, toads will actively disperse to rehydration sites (margins of water bodies and moist microhabitats in human habitation; Carpenter and Gillingham 1987; Child, Phillips, and Shine 2008, 2009; González‐Bernal et al. 2015)
3. *Behavioural Responses*—On each day, toads are either active (breeding, rehydrating, or foraging) or inactive in diurnal shelters. Only adult toads breed during the wet season, with males breeding multiple times and females only until they spawn (Hearnden 1991). Dehydrated toads can rehydrate from their immediate environment during the wet season, but they will have to migrate to water bodies to rehydrate during the dry season (Carpenter and Gillingham 1987; Child, Phillips, and Shine 2008; Child, Phillips, and Shine 2009; González‐Bernal et al. 2015). Active, non‐breeding and non‐rehydrating simulated toads are foraging (González‐Bernal et al. 2015).
4. *Reproduction*—Breeding toads select optimal spawning sites to maximise offspring survival, avoiding areas with many tadpoles (Clarke, Shine, and Phillips 2019; Hearnden 1991; McCann et al. 2020). A breeding female toad can lay 7675–14,288 eggs, based on its age/size (Hearnden 1991).
5. *Foraging Ecology*—Tadpoles forage along coastlines of spawning areas, whereas terrestrial toads forage in grassland and human inhabited areas (González‐Bernal et al. 2015). It is assumed that cane toads can always meet their minimum daily energetic needs while foraging.
6. Dispersal movement—the dispersal movement ability of juvenile and adult cane toads markedly varies between the wet and dry seasons. During the wet season, toads move further, move in straighter paths, and are more likely to change shelter‐sites on successive nights. Meanwhile, during the dry season, toadsare more likely to move in a random direction and are less likely to change shelter sites on successive nights. Cane toads prefer to disperse along roads, and the maximum distance they can travel in a day is about 1,000 m (rare long‐distance dispersal events).
7. *Mortality*—Toads have a chance of dying every day, with probabilities dependent on their life stage (eggs have the highest mortality rates), cannibalism of congeners during the egg stage and their water balance during the terrestrial life phases (Brown, Kelehear, and Shine 2011; Crossland et al. 2011; Hearnden 1991; Jørgensen 1991).

### 2.4 Computer Code and Software

*virToad* was developed and is run in HexSim (v.4.0.2; www.hexsim.net; Schumaker and Brookes 2018), which is a flexible platform for developing models with a broad range of ecological processes. *virToad* is open‐source and its code can be accessed at https://github.com/armanpili/virToad.

### 2.5 Model Output Verification and Corroboration

The basis of *virToad*’s usefulness in understanding species colonisation is its ability to simulate at a high degree of realism cane toads’ spatiotemporal population dynamics. Aside from the aforementioned life history processes, *virToad* reproduces several important patterns at population‐level:

1. The average adult population size of simulated toads along shorelines of water bodies (100 m buffer distance) is 398.61 per km of shoreline (95% CI = 396.03, 401.18), with the highest during the late‐dry period (516.88/km shoreline [95% CI = 513.46, 520.30]) followed by a gradual decrease until the wet‐to‐dry transition period (325.09/km shoreline [95% CI = 322.92, 327.25]; Smart, Tingley, and Phillips 2020).
2. The distribution of the population changes over time, owing to individual behavioural responses to landscape moisture. Specifically, the distribution of toads ‘pulses’ throughout the landscape, whereby all individuals are restricted near water bodies during the dry season and juveniles and adult females disperse throughout the landscape during the wet season (Phillips et al. 2007).
3. Seasonal sex bias throughout the landscape, whereby adults along shorelines of water bodies comprise 93.8% (min = 74.4%; max = 97.25%) males during the wet season. This sex bias gradually decreases to an average of 61.7% (min = 56.58%; max = 90.12%) male bias in the dry season (Hearnden 1991).
4. Breeding activity reaches its peak during the early‐wet season, following an ‘explosive’ breeding pattern (Hearnden 1991; Yasumiba, Alford, and Schwarzkopf 2016).
5. About 23.8% (min = 11.05%; max = 32.17%) of the cane toad population can be detected along shorelines of water bodies (i.e., detection probability) at any day during the wet season, which gradually decreases to 11.34% (min = 3.9%; max = 14.58%) in the dry season (Muller and Schwarzkopf 2017; Smart, Tingley, and Phillips 2020).

### 2.6 Parameter Sensitivity Analysis

The life history parameters ‘terrestrial phase growth rate’, followed by ‘adult survival rate’ and ‘tadpole development days’, had the most profound effect on the simulated toads' adult population growth and size. Interestingly, the distribution of adult females and adult males during the dry season was also sensitive to parameters related to water ecophysiology, specifically the ‘range of water sensing ability’ and ‘rate of water loss’.

## 3 Simulating Incursion Scenarios

### 3.1 Simulation Landscape and Environment

#### 3.1.1 Spatial and Temporal Resolution and Scale

**FIGURE 1:**
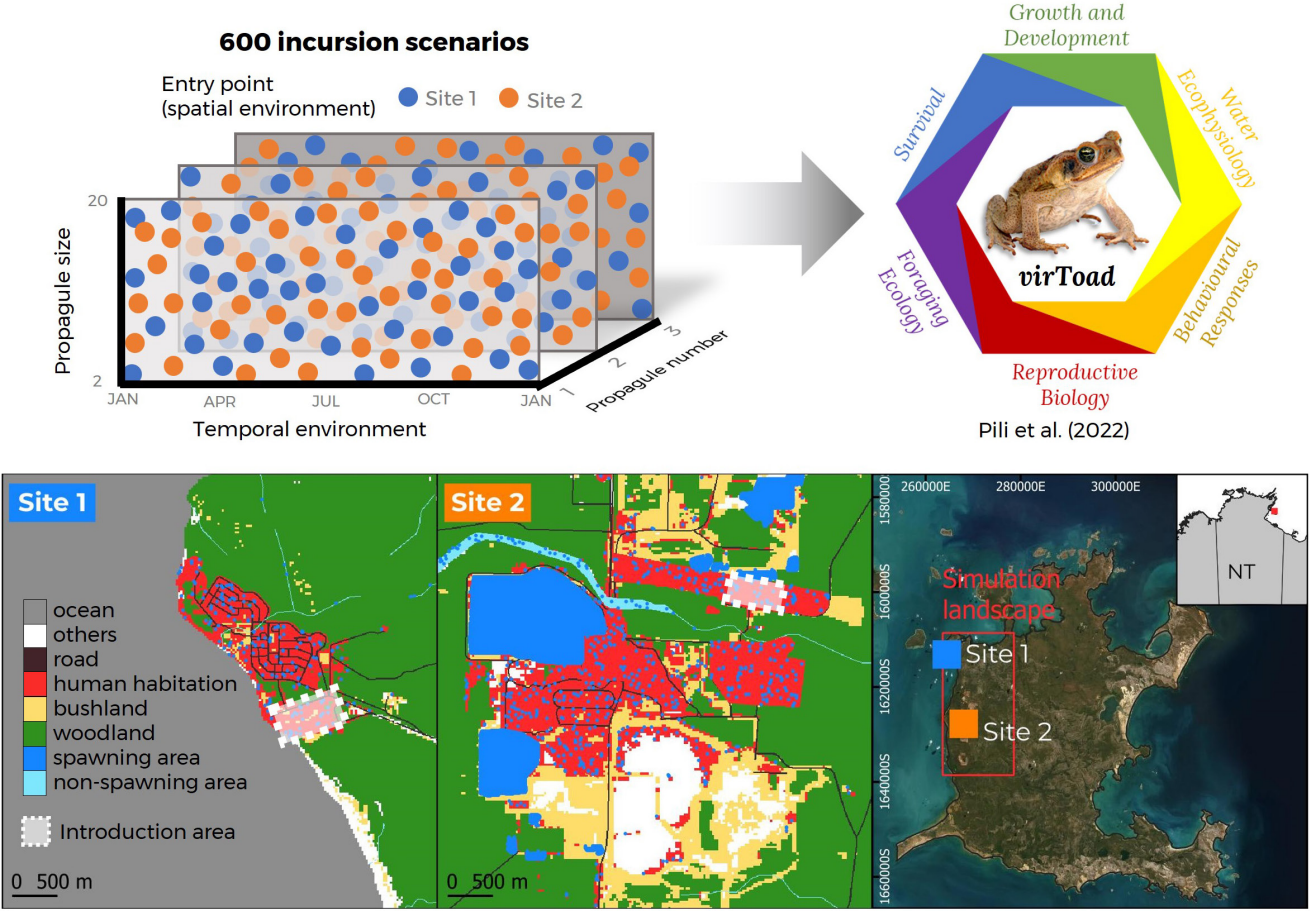
Simulating cane toad incursion scenarios using *virToad*. Under a latin hypercube sampling design, we sampled 600 parameter combinations (replicated 30 times, stimulating a total of 18,000 incursion scenarios) from a parameter hyperspace defined by propagule size (2–20 individuals), propagule number (one to three introduction events), spatial environment (two entry points) and temporal environment (initial introduction from Day one to 730). The simulation landscape is a 15 km × 30 km area in the western Groote Eylandt, Northern Territory, Australia (13.9667 S, 136.5833 N). It encompasses the main humaninhabited areas and socio‐economic hubs on the island (labelled Site 1 and Site 2).

We simulated cane toad incursion in a landscape with a 15 km by 30 km area (Figure 1). The simulation landscape comprises a two‐dimensional array of 86.6 m^2^ hexagonal cells, with the distance between hexagon centres being 10 m (total of 5,196,000 hexagon cells). We ran incursions with a 1‐day time step and up to 3 years. Our preliminary results indicated no substantial difference in probabilities of cane toad establishment when run for more than 3 years (i.e., 5, 10 and 15 years).

#### 3.1.2 Spatial and Temporal Environment

We characterised the simulation landscape and environment based on the spatial distribution of habitats and dynamic rainfall conditions in west Groote Eylandt, Northern Territory, Australia (13.9667° S, 136.5833° N). Groote Eylandt is the fourth largest island in Australia (~2326.1 km^2^). It is located approximately 50 km east of the Northern Territory mainland, in the Gulf of Carpentaria. It has a tropical savannah wet‐dry climate, where the wet season lasts from November to April. Groote Eylandt's landscape is structurally heterogeneous, broadly classified into ocean, road, human habitation, bushland, woodland, ephemeral free‐flowing waterbody, non‐flowing waterbody and other habitat types.

Groote Eylandt is currently cane toad‐free but at high risk of invasion by cane toad (Smart, Tingley, and Phillips 2020). Since 2008, dead and alive cane toads have been intercepted in Alyangula township (Figure 1, Site 1) and Angurugu community (Figure 1, Site 2; Spring and Kompas 2017). These are the island's two most densely human‐populated areas and where major transport hubs are found (Groote Eylandt Mining Company Operation [GEMCO] port ship loader and Groote Eylandt Airport). Our simulation landscape encompasses these areas.

- Site 1—Alyangula township (13.8490° S, 136.4195° N). The Alyangula is the largest township in Groote Eylandt with a human population size of about 870. The landscape of Alyangula is characterised by a single ‘packed’ patch of human‐disturbed habitats (4 km^2^ area of human housing, resorts, golf courses and public infrastructure, including a wharf) within a matrix of woodland habitats.
- Site 2—Angurugu community (13.9735° S, 136.4576° N). Site 2 is where GEMCO operates its mining activities. Its landscape comprise of fragmented human‐disturbed and open‐water habitats (about 9.7 km^2^ area of infrastructures, roads, exposed soils and several pit lakes) within a matrix of woodland habitats.

### 3.2 Incursion Scenarios

Incursion scenarios varied in the following initialisation parameters:

1. *Timing of arrivals*—We introduced simulated toads between Days 0 and 730. The simulation has a temporally dynamic environment that emulates the daily rainfall patterns in Groote Eylandt.
2. *The entry point of arrivals*—We introduced simulated toads in two entry points. The simulation landscape emulated the spatial heterogeneity of habitats of two known entry points in Groote Eylandt—a wharf and an airport (Figure 1, Sites 1 and 2).
3. *Number of arriving individuals* or *propagule size*—We introduced 2–20 toads at each introduction event.
4. *Frequency of arrival events* or *propagule number*—We introduced toads in one or multiple (2 or 3) introduction events.

As an improvement to previous simulation‐based studies (e.g., Cassey, Prowse, and Blackburn 2014), cane toad introduction events in our simulations are spatially and temporally overlapping/non‐independent—that is, cane toads can survive between separate introduction events and cane toads introduced in separate introduction events can interact (i.e., breed) with each other.

#### 3.2.1 Initial Parameters

The four aforementioned initialisation parameters result in 7 × 10^9^ possible parameter combinations (i.e., parameter hyperspace). For practicality reasons, we sampled 600 parameter combinations from this parameter hyperspace using a Latin hypercube sampling design (using ‘lhs’ package v.3.4.0. in *R* statistical platform; Carnell 2022; R Core Team 2023). Here, parameter combinations are sampled at near‐random from a multidimensional distribution while ensuring that the final set of parameter combinations is uncorrelated and representative of each parameter's real variability (McKay, Beckman, and Conover 2000; Stein 1987). Furthermore, we optimised the sampling design using an ‘optimum’ algorithm, which maximises the mean and minimum distance between each parameter combination in parameter hyperspace (Stein 1987).

#### 3.2.2 Other Miscellaneous Initial Parameters

On introduction, simulated toads have an equal chance (50%:50%) of being male or female. Toads also have a starting snout‐to‐vent length (SVL; i.e., size) of 90 mm on average (~70 to 110 mm). This is the same sex ratio and SVL of intercepted cane toads (White and Shine 2009).

#### 3.2.3 Simulating Incursion Scenarios

We run each incursion scenario for 3 years from the first introduction or until no cane toads remain in the simulation, whichever comes first. We run each parameter combination with 30 replicates for a total of 18,000 incursion scenario simulations. For each replicate, we considered the cane toad population ‘established’ (i.e., 0 = failed, 1 = success) if their population growth rate (adults only) increased at the end of the simulation (i.e., $\frac{\text{final adult population size}}{\text{total introduced individuals}}>1.0$).

### 3.3 Statistical Analysis

#### 3.3.1 Generalised Linear Modelling

By fitting simulation outputs to generalised linear models with a binomial function (i.e., bGLMs; using base ‘stats’ package v.3.6.2.; Hosmer, Lemeshow, and Sturdivant 2013; R Core Team 2023), we modelled the cane toad's establishment probability as a function of the individual and interacting effects of propagule size, propagule number and/or spatial environment. We first employed the original method of quantifying establishment probability described by Cassey et al. (2018). Here, we fitted bGLMs with only propagule size as a predictor variable (bGLM‐1). We then build on this model by adding two predictor variables and their interactions: propagule number (bGLM‐2) and, subsequently, the spatial environment (bGLM‐3).

**FIGURE 2:**
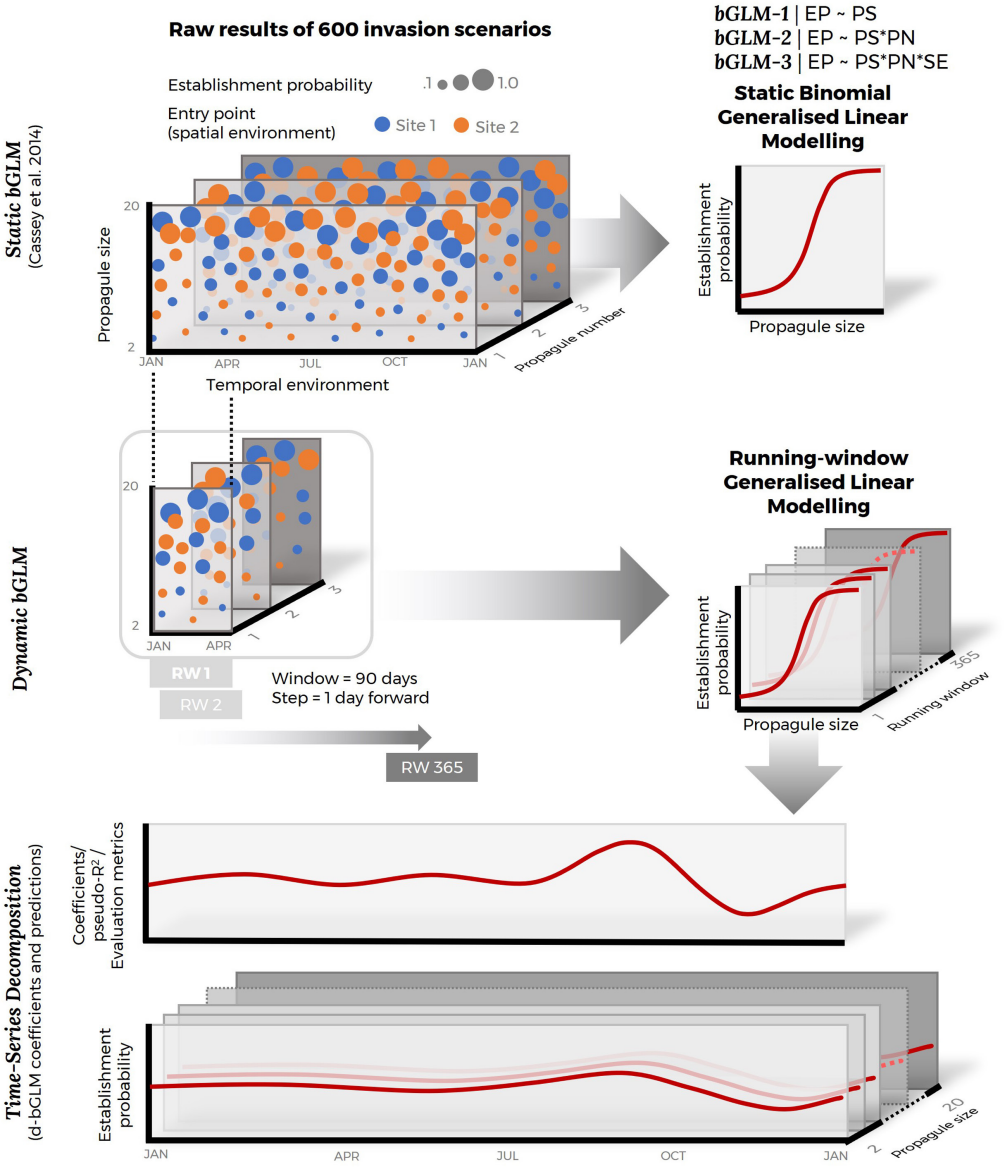
Statistical analysis workflow. Generalised linear models with binomial function (bGLM) were fitted using simulation outputs. The simplest bGLM (bGLM‐1) modelled the effect of propagule size (PS) on relative establishment probability (RER). We then build on bGLM‐1 by adding the interacting effects of propagule number (PN; bGLM‐2) and then spatial environment (SE; bGLM‐3). To assess the effect of temporal environment, we fitted 365 bGLM‐3 with 90‐day running window (1‐day step) of simulation outputs (rw‐bGLM‐3). Finally, we extracted, collated into a time series and visualised changes in time of the coefficients and predictions of the rw‐bGLMs.

We developed two sets of bGLMs. The first set of models is fitted with the entire simulation outputs and does not account for the effects of the timing of introduction. We call these static bGLMs or ‘s‐bGLMs’. To account for the effects of the timing of introduction, we developed a second set of models. These comprise a time series of bGLMs, each fitted with a running window of simulation outputs (described below). We call these models dynamic bGLMs or ‘d‐bGLMs’. See Figure 2 to visualise the modelling workflow.

For d‐bGLMs, we first ordered the simulation outputs by the timing of introduction (or first introduction for the case of multiple introduction events). We then iteratively fitted bGLM‐1, bGLM‐2 and bGLM‐3 on a 90‐day running window, with 1‐day forward step of simulation outputs. For instance, we fitted a set of bGLMs (bGLM‐1, bGLM‐2 and bGLM‐3) with simulation outputs whose timing of introduction was between Days 1 and 90 (1 January to 31 March). We can interpret these bGLMs as the establishment probability on the 45th day of the year (14 February; the centre of the window). We then moved the window forward by 1 day to Days 2–91 (2 January to 1 April) and used this to fit another set of bGLMs. We repeated this process until we had a time series of bGLMs depicting the establishment probability on each day of the year; or more specifically, 365‐day time series bGLM‐1s ‘(“d‐bGLM‐1”’), bGLM‐2s ‘(“d‐bGLM‐2”’) and bGLM‐3s ‘(“d‐bGLM‐3”’).

We plotted the predictions of d‐bGLMs to analyse how establishment probability changed over time. Finally, we visually inspected if the predictions of the s‐bGLMs were different (higher or lower) from the predictions of d‐bGLM‐3 at any point in time. Although we are more interested in magnitude of difference (i.e., effect size), we also visually inferred statistical significance based on the rule that an overlap of half the length of one 95% confidence interval arm corresponds approximately to a statistical significance at *p* = 0.05 (Cumming 2009).

#### 3.3.2 Model Evaluation

We evaluated the relative performance of bGLMs by analysing the distribution of residuals, goodness‐of‐fit and predictive performance (Gelman and Hill 2006; Hosmer, Lemeshow, and Sturdivant 2013).

We inspected the distribution of residuals through a binned residual plot (using ‘arm’ package v.1.12 in R; Gelman et al. 2013). Here, data are first divided into bins based on fitted values. Then, we plotted the average residual values versus the average fitted value of each bin. From this, a model is reasonable if most of the fitted values fall within the 95% confidence interval of observed residual values.

We evaluated the models' goodness‐of‐fit by computing McFadden's *R* ^2^. McFadden's *R* ^2^ measures the improvement of the model over a null model with only the intercept as a predictor. Its values fall between 0 to just under 1, with values closer to 0 indicating that the model has no predictive power (Hosmer, Lemeshow, and Sturdivant 2013).

We evaluated the models' predictive performance through a 10‐fold cross‐validation approach (Stone 1974). Here, the simulation output is randomly partitioned into 10 equally sized ‘k’ folds. One k‐fold is held out as the validation data for testing the model, while the other *k*‐1 folds are used to train the model. The model is then used to predict the held‐out testing data, and its predictive performance is measured by computing accuracy, precision, recall, F1‐score and the area under the receiver operating characteristic curve (AUC; Hosmer, Lemeshow, and Sturdivant 2013). This process is repeated 10 times, with each *k* subsamples used once as the validation data.

Accuracy indicates the proportion of observed values equal to the predicted values. Precision measures the proportion of observed values predicted positive (i.e., equals 1) that are actually positive. Recall measures the proportion of actual positives that were correctly predicted as positive. F1 score is the harmonic mean of precision and recall. The AUC measures the probability that a randomly chosen observed value that is actually positive has a higher predicted value than a randomly chosen observed value that is actually negative (equals 0). We interpreted AUC values based on Swets (1988), where values > 0.90 = excellent, > 0.80–0.90 = good, > 0.70–0.80 = fair, > 0.60–0.70 = poor and > 0.50–0.60 = fail.

We evaluated if adding the effects of propagule number and spatial environment significantly improved predicting establishment probability. We did this by comparing the residual deviance and the sum of squared residuals of the three sets of bGLMs using analysis of variance (ANOVA; Chambers, Freeny, and Heiberger 2017). The comparison was done separately for s‐bGLMs and d‐bGLMs. We also performed Tukey's honest significant difference test on bGLM‐3 and d‐bGLM‐3 to identify which groups' means are significantly different (Tukey 1949).

For d‐bGLMs, we compiled a 365‐day time series of McFadden's *R* ^2^, cross‐validation evaluation metrics, residual deviance and the sum of square residuals. We visualised the time series to assess how bGLM performance changed over time.

## 4 Results

Because of the similarity in the patterns of their results, we use ‘bGLM‐1’, ‘bGLM‐2’ and ‘bGLM‐3’ to refer to both static and dynamic bGLM‐1, bGLM‐2 and bGLM‐3, respectively, unless explicitly specified.

### 4.1 Model Performance

Binned residual plots revealed that bGLM‐3 had the best fit for simulation outputs (see Figure S1). All models tend to overpredict fitted establishment probability values below 0.3. However, bGLM‐1 and bGLM‐2 struggled in (underpredicted) fitting establishment probability values between 0.4 and 0.6.

McFadden's *R* ^2^ and cross‐validation metrics revealed that bGLM‐3 consistently performed much better than bGLM‐2, which was only slightly better than bGLM‐1 (Table 1). McFadden's *R* ^2^ indicates that all models have better predictive performance than a null model. Similarly, AUC scores indicate that all models have good predictive performance (> 0.8). Finally, accuracy and F1 indicate that all models were quite accurate (85%–87%) in correctly predicting successfully established scenarios, and fairly accurate (79%–81%) in correctly predicting scenarios that successfully established or failed to establish.

**TABLE 1:** Predictive performance of static and dynamic bGLMs. bGLMs modelled the individual and/or interacting effects of propagule size (PS), propagule number (PN) and spatial environment (SE) on establishment probability (EP). Their goodness‐of‐fit was evaluated by computing McFadden's *R* ^2^. Meanwhile, their predictive performance was measured under a 10‐fold cross‐validation approach and summarised using the metrics area under the receiver operating characteristic curve (AUC), accuracy, precision, recall and F1. For dynamic bGLMs, the mean and standard deviation of the evaluation metrics of 365 bGLMs are shown.

Model	bGLM	McFadden's *R* ^2^	AUC	Accuracy	Precision	Recall	*F*1
Statis bGLMs							
s‐bGLM‐1	EP~PS	0.273	0.841	0.792	0.876	0.838	0.857
s‐bGLM‐2	EP~PS*PN	0.275	0.841	0.792	0.876	0.838	0.857
s‐bGLM‐3	EP~PS*PN*SE	0.351	0.878	0.811	0.901	0.836	0.868

Model	bGLM	McFadden's *R* ^2^ (SD)	AUC (SD)	Accuracy (SD)	Precision (SD)	Recall (SD)	*F*1 (SD)
Dynamic bGLMs							
d‐bGLM‐1	EP~PS	0.275 (0.034)	0.841 (0.27)	0.792 (0.024)	0.878 (0.022)	0.835 (0.30)	0.856 (0.019)
d‐bGLM‐2	EP~PS*PN	0.279 (0.033)	0.842 (0.027)	0.79 (0.026)	0.880 (0.022)	0.83 (0.033)	0.854 (0.021)
d‐bGLM‐3	EP~PS*PN*SE	0.353 (0.031)	0.877 (0.022)	0.812 (0.022)	0.898 (0.018)	0.842 (0.029)	0.869 (0.017)

**FIGURE 3:**
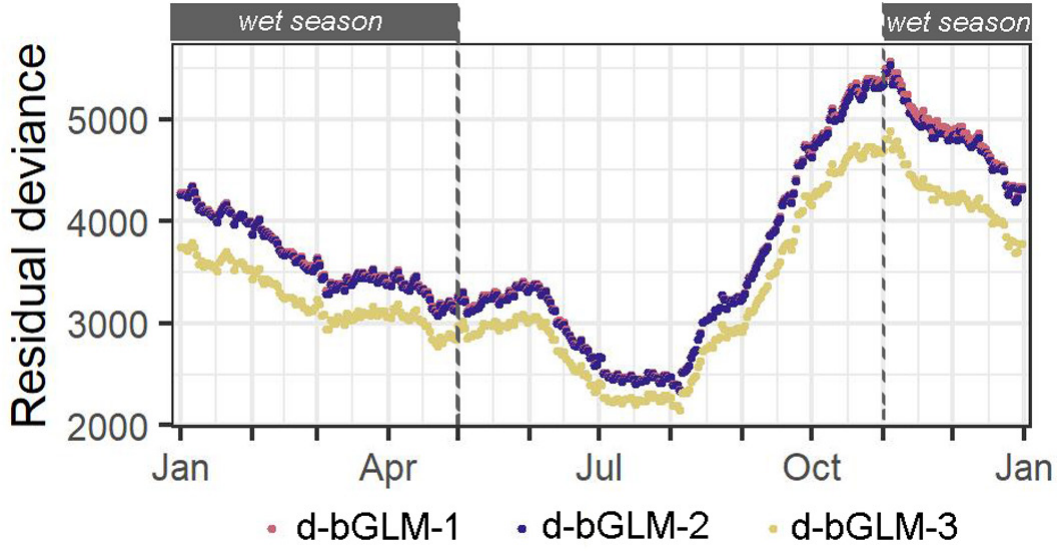
Temporal trend of goodness‐of‐fit of dynamic bGLMs. bGLMs modelled the individual and/or interacting effects of propagule size, propagule number and spatial environment on establishment probability. The residual deviance of running‐window (90‐day window) bGLMs were compiled into a 365‐day time series, which we plotted to compare goodness‐of‐fit with increasing model complexity (d‐bGLM‐1, d‐bGLM‐2 and d‐bGLM‐3) and to assess how goodness‐of‐fit changes in time.

The mean McFadden's *R* ^2^ and cross‐validation evaluation metrics indicate that d‐bGLMs only slightly performed better than their s‐bGLM counterparts. Notably, the performance of d‐bGLMs fluctuated in time, wherein bGLMs during the mid‐dry season performed best, and those during the late‐dry to early‐wet season performed worst (Figure 3; see also Figure S1).

Analysis of variance showed that adding the individual and interacting effects of propagule number (bGLM‐2) and spatial environment (bGLM‐3) increased the predictive performance of models (Table 2; see also Figure S1). However, upon inspecting time series residual deviance and the sum of squared residuals values of d‐bGLM‐2, we found that the individual and interacting effects of propagule number were only substantial and significant in 290 of 365 d‐bGLMs. Meanwhile, the individual and interacting effects of spatial environment were substantial and significant in all d‐bGLM‐3.

**TABLE 2:** The goodness‐of‐fit of static and dynamic bGLMs. bGLMs modelled the individual and/or interacting effects of propagule size (PS), propagule number (PN) and spatial environment (SE) on Establishment probability (EP). Analysis of variance test revealed that an increase in model complexity results in a statistically significant improvement in goodness‐of‐fit.

Model	Predictors	df	Residual deviance	Deviance	*F*	*F* < Pr
Static bGLMs						
s‐bGLM‐1	EP~PS	1	15,120			
s‐bGLM‐2	EP~PS*PN	5	15,068	51.76	12.941	*
s‐bGLM‐3	EP~PS*PN*SE	11	13,494	1573.87	252.311	*

Model	Predictors		Residual deviance (SD)	Deviance (SD)	*F* (SD)	*F* < Pr (no. models)
Dynamic bGLMs						
d‐bGLM‐1	EP~PS	1	3763.613 (861.339)			
d‐bGLM‐2	EP~PS*PN	5	3742.246 (849.395)	21.367 (15.661)	5.342 (3.915)	* (290/365)
d‐bGLM‐3	EP~PS*PN*SE	11	3348.442 (719.478)	415.171 (146.817)	14.682 (14.681)	* (365/365)

* *p* value < 0.05.

**FIGURE 4:**
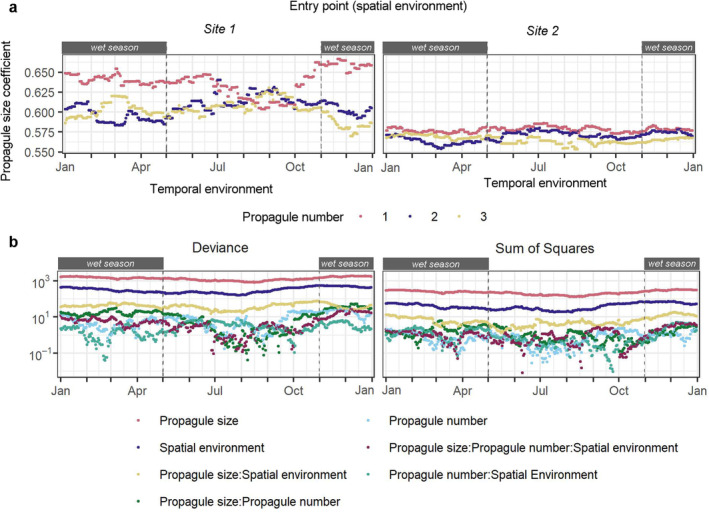
Temporal trend in (a) the regression coefficient of propagule pressure and (b) the amount of variation explained by predictor variables of d‐bGLM‐3.

In both s‐bGLM‐3 and d‐bGLM‐3, propagule size consistently explained the most variation in models, followed by spatial environment and the interaction between propagule size and spatial environment (Table 3, Figure 4). Notably, in d‐bGLM‐3, the propagule number alone or its interaction with other predictors consistently explained low variation in models and was not significant in most models.

**TABLE 3:** The amount of variation explained by individual predictor variables or interactions between predictor variables of bGLMs. bGLMs modelled the individual and/or interacting effects of propagule size (PS), propagule number (PN) and spatial environment (SE) on establishment probability (EP).

Parameter	Deviance	Pr > chi square	Sum squares of residuals	*F* < Pr
s‐bGLMs‐3				
Propagule size	5666.9	*	962.8	*
Spatial environment	1373.3	*	171.1	*
Propagule size: Spatial environment	173.4	*	26.4	*
Propagule size: Propagule number	43.7	*	3.2	*
Propagule number	15	*	0.1	
Propagule size: Propagule number: Spatial environment	14.5	*	1.6	*
Propagule number: Spatial environment	5.7		3	*

Parameter	Deviance (SD)	Pr > chi square (prop. of bGLM‐3 s)	Sum squares of residuals (SD)	*F* < Pr (prop. of bGLM‐3 s)
d‐bGLMs‐3				
Propagule size	1412.483 (12.536)	(365/365)*	240.172 (59.030)	(365/365)*
Spatial environment	340.373 (121.978)	(365/365)*	42.791 (16.824)	(365/365)*
Propagule size: Spatial environment	42.144 (14.273)	(365/365)*	6.797 (4.007)	(365/365)*
Propagule size: Propagule number	14.121 (12.536)	(247/365)*	1.596 (1.214)	(251/365)*
Propagule number	9.121 (7.886)	(200/365)*	0.875 (0.589)	(187/365)*
Propagule size: Propagule number: Spatial environment	6.273 (6.037)	(136/365)*	1.308 (1.117)	(223/365)*
Propagule number: Spatial environment	2.85 (1.823)	(30/365)*	1.184 (1.198)	(163/365)*

* *p* value < 0.05.

### 4.2 Predictions

**FIGURE 5:**
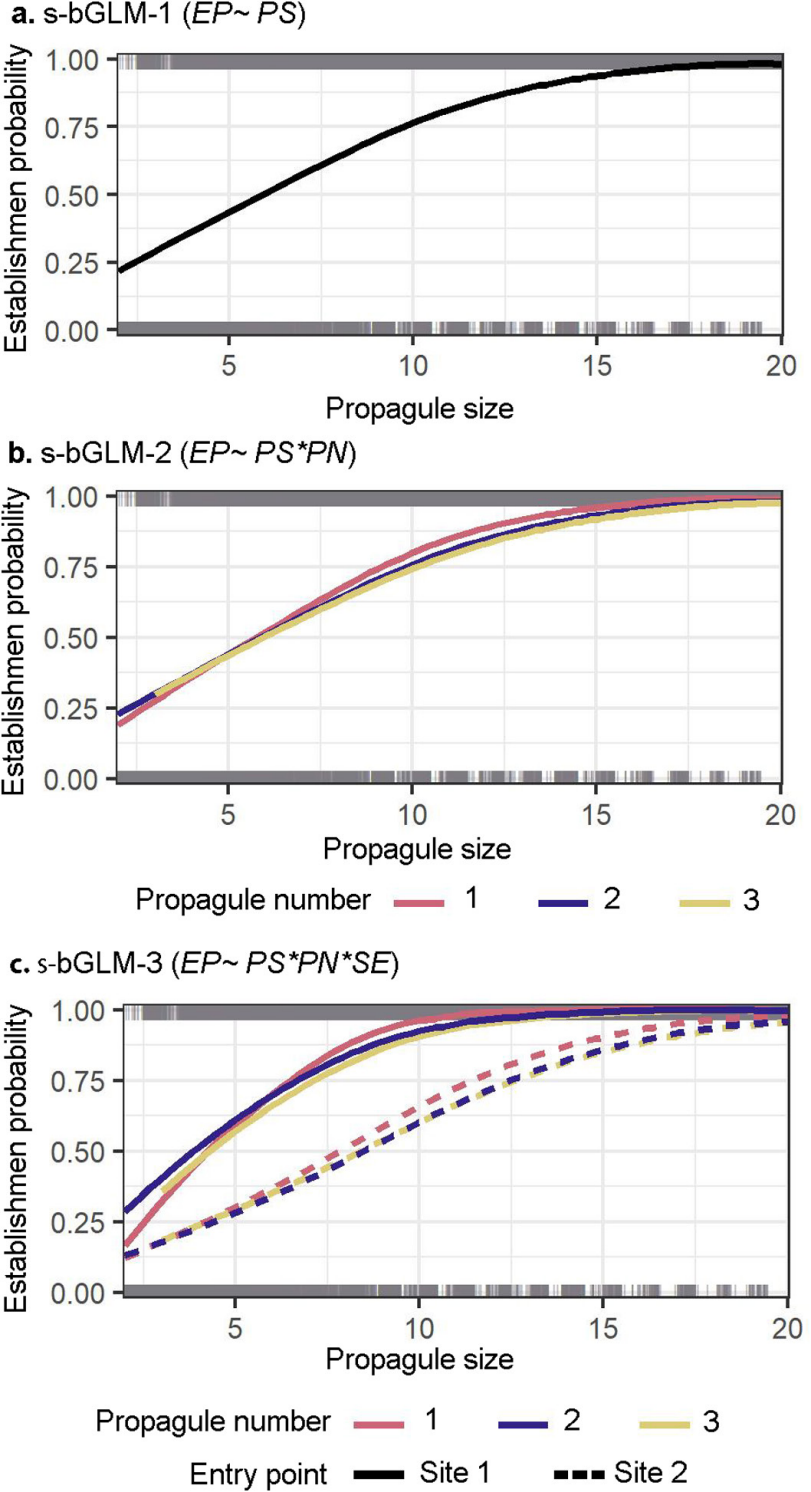
Establishment probability (EP) of cane toads in Groote Eylandt predicted by static bGLMs: (a) s‐bGLM‐1, (b) s‐bGLM‐2 and (c) s‐bGLM‐3. The bGLMs modelled the individual and/or interacting effects of propagule size (PS), propagule number (PN) and spatial environment (SE) on EP.

The holistic patterns of predicted establishment probability coincide with the patterns inferred from models' relative performance and the relative importance of predictor variables. The predicted establishment probability of bGLM‐2 is slightly, but statistically non‐significantly, lower than that of bGLM‐1. Meanwhile, predictions of bGLM‐3 are statistically significantly higher (Site 1) or lower (Site 2) than those of bGLM‐1 and bGLM‐2 (Table 2, Figure 5).

**FIGURE 6:**
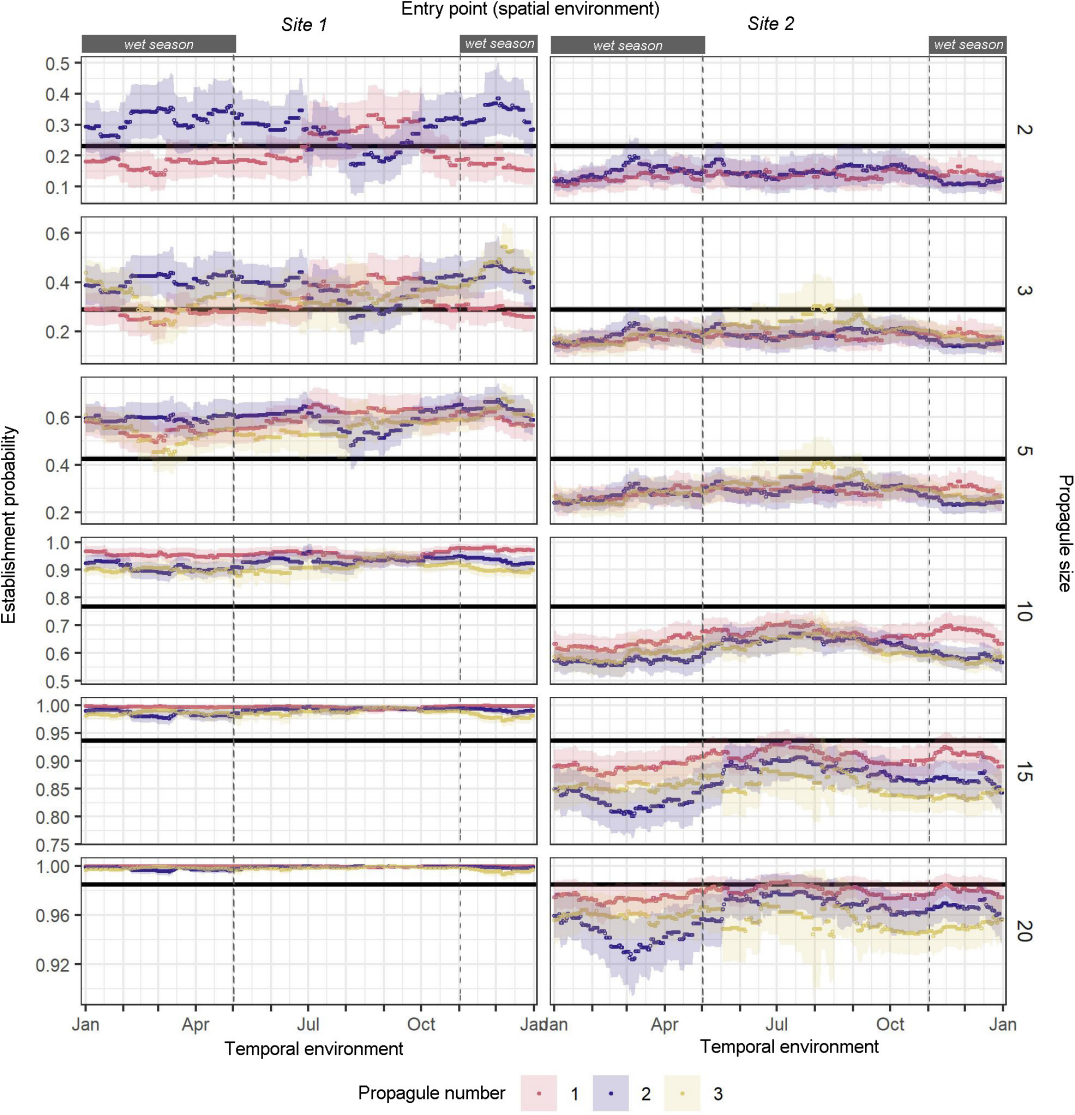
Temporal trend in establishment probability (EP) of cane toads in Groote Eylandt predicted by d‐bGLM‐3 (coloured points) and s‐bGLM‐1 (horizontal black line). The d‐bGLM‐3 modelled the individual and interacting effects of propagule size (PS), propagule number (PN) and spatial environment (SE) on EP. Ribbons indicate the 95% confidence intervals of predictions.

To understand more comprehensively the interacting effects of predictors in time, we further investigated how the establishment probability of s‐bGLM‐1 and d‐bGLM‐3 would change along an increasing gradient of propagule size and in time (Figures 6).

First, introducing two to three cane toads on separate introduction events (the blue and yellow lines and bands in Figure 6) would increase establishment probability by up to 15%. But this pattern was not consistent over time; during the late‐dry season, two to three cane toads introduced simultaneously (the red lines and bands in Figure 6) would have up to 12.5% higher establishment probability than if introduced separately. Conversely, consistent over time, introducing five or more cane toads on separate introduction events slightly decreases or has a negligible effect on establishment probability.

Second, cane toads have a higher probability of establishing in Site 1 than in Site 2. This is consistent over time. For instance, introducing two cane toads has a 32% establishment probability in Site 1, but only 12.5% in Site 2. Meanwhile, introducing 10 cane toads has a 90% establishment probability in Site 1, but up to 75% in Site 2. Finally, incursion scenarios involving 20 or more cane toads, regardless of whether they were introduced on the same introduction event, in different entry points, and/or at any point in time, have > 90% establishment probability.

Comparing predictions of s‐bGLMs versus d‐bGLM3, we found that the predicted establishment probability of s‐bGLM1 (Figure 6) and s‐bGLM2 (see Figure S1) tend to be statistically significantly different (lower and higher) than the predictions of d‐bGLM3 across increasing propagule size, different propagule number, different entry points and in time. Meanwhile, the predicted establishment probability of s‐bGLM3 is seldom statistically significantly different from predictions of d‐bGLM3 (see Figure S1).

## 5 Discussion

Propagule pressure and the environment play crucial roles in population establishment and, by extension, species colonisation. Nonetheless, in small founding populations, typifying most natural and human‐mediated colonisation events, the large variability in establishment probability has been frequently dismissed as context‐dependent (Cassey et al. 2018). That is, the variation in magnitude and signs of relationships of establishment probability and propagule pressure vary in different spatial, temporal, demographic, environmental or observational circumstances (Catford et al. 2022; Caughley 1994; O'Reilly‐Nugent et al. 2016; Pickett and Cadenasso 1995). However, the widespread failure to understand the sources of this context dependence undermines the generality and predictability of establishment probability (Catford et al. 2022; Elliott‐Graves 2015). Here, we shed light on how two putative and overlooked sources of context dependence—(1) landscape spatial heterogeneity and (2) temporally dynamic environmental conditions, interplay with propagule pressure in establishing small founding populations. By simulating incursion scenarios of a model invasive alien species, as expected, [Hypothesis 1] we found a strong positive relationship between establishment and propagule size, whereby establishment increases with increasing number of founding individuals. Meanwhile, propagule number had a weak effect (Tables 2 and 3). Nonetheless, establishment success was highly variable in realistic incursion scenarios (Figure 6), such as small founding populations with five or fewer individuals (Pili et al. 2023). Upon deeper analysis of this variability, we found support for landscape spatial heterogeneity and dynamic environmental conditions as two compelling sources of this context dependence (Table 3 and Figures 5 and 6). Small founding populations' establishment success tends to peak in packed landscapes (Site 1) where there are relatively limited and clustered habitats for movement, survival, and reproduction [Hypothesis 2] and when individuals are introduced shortly before the breeding season (Hypothesis 3; up to 74% establishment success rate compared to 58% annual mean; Figure 6). Herein, we discuss the putative mechanistic underpinnings of why landscape spatial heterogeneity and dynamic environmental conditions influence establishment success. We end by discussing how accounting for these sources of context dependence is imperative in designing, interpreting and communicating applied biogeography research, particularly in reintroduction biology and invasion science.

In environmentally suitable areas, propagule pressure primarily drives species establishment and, by extension, colonisation (Cassey et al. 2018; Deredec and Courchamp 2007). Our findings fit well with Cassey et al.'s (2018) global and cross‐taxonomic review that establishment probability and confidence of predictions increase with the number of founding individuals (i.e., propagule size), regardless of whether they arrived together or in separate dispersal events (i.e., propagule number). Contrarily, we also found propagule pressure alone weakly predicts the establishment of small founding populations. Our simulations of cane toad incursions showed near‐certain and highly confident predictions of the establishment of founding populations comprising 20 or more individuals. This indicates that, at this level, propagule pressure swamps the effects of other confounding factors—for example, demographic and environmental stochasticity. But, at smaller propagule sizes, establishment success drastically loses confidence—a pattern also similar to the findings of Cassey et al. (2018). This concerning uncertainty in establishment rate has been attributed to context dependence (Catford et al. 2022) or even so due purely to chance. And our study investigated the underlying sources of context dependence in small founding populations' establishment process.

The composition and configuration of habitats in a landscape—that is, landscape spatial heterogeneity, is a fundamental causal factor driving processes across ecological scales (O'Reilly‐Nugent et al. 2016; Pickett and Cadenasso 1995). With regard to the colonisation process, research attention has recently shifted towards understanding the influence of landscape spatial heterogeneity on species colonisation success (see review by O'Reilly‐Nugent et al. 2016). Species colonisation success would vary in different landscapes (see Brown et al. 2006; González‐Bernal et al. 2015; Ward‐Fear, Greenlees, & Shine, 2016); intuitively, species are more likely to colonise landscapes with relatively more suitable habitats for movement, survival and reproduction (O'Reilly‐Nugent et al. 2016; see sensitivity analysis of Pili et al. 2022; Walter, Johnson, and Haynes 2017). However, an exception to this premise is colonising species that start with small founding populations; several studies suggest small founding populations' establishment (or failure) is due to chance—that is, demographic and environmental stochasticity (Duncan et al. 2014; Melbourne and Hastings 2009). Our findings do not entirely agree: firstly, we found that landscape spatial heterogeneity can explain a significant variation in the establishment success of small founding populations; as revealed by the results of bGLM‐3 (see Table 3 and Figures 5 and 6), the establishment success in Site 1 is higher than in Site 2. Secondly, we found that higher proportions of habitats for movement, survival, and reproduction, as with the case in Site 2 (see Figure 1), do not necessarily facilitate, but may even inhibit, the establishment of small founding populations. In line with the Allee effect (Courchamp, Clutton‐Brock, and Grenfell 1999; Gascoigne et al. 2009), individuals of small founding populations are more likely to find a compatible mate in *packed* landscapes—that is, landscapes with small proportions and clustered configurations of key habitats for movement, survival, and reproduction, as with the case in Site 1 (see Figure 1; O'Reilly‐Nugent et al. 2016). Such landscapes restrict and confine individuals' movement, resulting in disproportionately higher spatial population densities in suitable habitats, which in turn facilitate individuals to meet in limited breeding sites (Deredec and Courchamp 2007; Gascoigne et al. 2009). Landscape spatial heterogeneity clearly plays a foremost role in the establishment of founding populations and, by extension, species colonisation. And this indispensable source of context dependence, among other crucial confounding factors such as dynamic environmental conditions, require priority consideration in planning, designing and interpreting biogeographical experiments or on‐the‐ground management strategies.

Dynamic environmental conditions influence the timing of many life history traits and behaviours (i.e., phenology), and it is equally as essential as landscape spatial heterogeneity in causing bottom‐up effects on ecological processes across space, time and scale (Forrest and Miller‐Rushing 2010; Pickett and Cadenasso 1995). As we have shown here, the population establishment of colonising species is no exception (see also March‐Salas and Pertierra 2020; Martin et al. 2014; Phillips et al. 2007; Sentis, Montoya, and Lurgi 2021). For many sexual species, the essential life history processes for colonisation, particularly dispersal and reproduction, have been evolutionarily synchronised, typically with regard to seasons of high ecosystem productivity (Calatayud, Stoops, and Durrant 2018; Frederiksen et al. 2004; Joly 2019; Segrestin et al. 2018). The dispersal and breeding phenologies of the cane toad in Australia exemplify this. Newly recruited adult cane toads along the invasion front are most dispersive right before the breeding season (late‐dry to early‐wet season; Phillips et al. 2007). The first to arrive in new areas would have a fitness advantage because their offspring dominate resources made bountiful by the wet season (Kelehear and Shine 2020). However, different climate regimes between origin and recipient (or sink and source) areas may require colonising species to shift or even lose synchrony of their phenologies (analogous to climate change‐induced shifts in phenologies; see Visser and Both 2005). Such shifts in phenologies would be most profound in trans‐global long‐distance dispersing species, which, in fact, is relatively common, as shown in the case of alien amphibians and reptiles arriving in New Zealand (Pili et al. 2023). We have demonstrated that unsynchronised dispersal (i.e., timing of introduction) and breeding phenologies can have significant implications for population establishment success, especially for small founding populations. Small founding individuals dispersing before or early in the period suitable for breeding (e.g., wet season for cane toads) are more likely to establish. In comparison, those arriving after the period suitable for breeding would mean delaying reproduction and surviving through to the next year, which ultimately is detrimental to population establishment. Because of its crucial role in synchronising life history processes, dynamic environmental conditions are a source of context dependence that cannot be dismissed when trying to understand and robustly predict establishment and colonisation of species, especially those that begin with small founding populations.

The widespread failure to identify and account for the sources of context dependence, much so its mechanistic underpinnings, in the establishment and colonisation of small founding populations leaves an impression that these ecological processes are not generalisable, transferable, nor predictable (Armstrong and Wittmer 2011; Buchadas et al. 2017; Catford et al. 2022; Cuddington et al. 2013; DeAngelis and Yurek 2016; Pickett and Cadenasso 1995). Moreover, because small founding populations exemplify most natural and human‐mediated species colonisation events (Deredec and Courchamp 2007; Pili et al. 2023; Wilson et al. 2009), this lack of understanding is problematic not just to theoretical but also applied biogeography, in general (Catford et al. 2022; Caughley 1994). By shedding light on two crucial sources of context dependence in the establishment of small founding populations, our study provides important insights for applied biogeography:

- For *species reintroduction programmes*, strategies should account for the composition and configuration of the release site landscape's habitats, especially those essential for species' movement, survival and reproduction. This is opposed to solely accounting for the ‘proportion’ of suitable habitats in the landscape (O'Reilly‐Nugent et al. 2016). Our findings suggest that releasing founding individuals in landscapes with *packed* essential habitats may increase establishment success. Packed landscapes confine animals into an area and provide limited options for sites where breeding individuals can meet (Armstrong and Wittmer 2011; Deredec and Courchamp 2007; Gascoigne et al. 2009). As such, packed landscapes may help mitigate the Allee effect—specifically, mate limitation (Gascoigne et al. 2009), by reducing post‐release dispersal and making individuals find compatible mates easier (Deredec and Courchamp 2007; see Molles et al. 2008 for strategies with similar premise; Stephens and Sutherland 1999). Nonetheless, species reintroduction strategies should also account for phenologies of key life history processes, particularly dispersal and reproduction phenologies, as well as how these phenologies are cued by and synchronised with regards to dynamic environmental conditions. Our findings suggest that releasing individuals before or early into periods suitable for breeding would significantly increase establishment success.
- In *assessing the invasion* probability of sites to unwanted biological invasions (i.e., site susceptibility; McGeoch et al. 2015), the composition and configuration of habitats in the landscape of alien species entry points should be taken into account. Following our findings, entry points with *packed* landscapes are more susceptible to alien species invasions (O'Reilly‐Nugent et al. 2016). A counterintuitive landscape management strategy would be to reduce available key habitats, especially breeding habitats. By doing this, species are deceived into concentrating on a location where management efforts should be prioritised (González‐Bernal et al. 2015, 2016; Gonzalez‐Bernal et al. 2012). Moreover, because alien species can potentially arrive at any time, the most conservative strategy would be to schedule management efforts year‐round but heighten efforts right before and during periods suitable for the species to breed (supporting experimental findings of Pili et al. 2022).

Importantly, the generalisability of our findings should be interpreted with caution. Given that our simulations are based on the biology and ecology of the cane toad, our results are most applicable to anthropophilic, highly mobile, semi‐aquatic species that breed seasonally in open‐water habitats. These life history and ecological attributes may explain why we found a higher importance of packed landscapes and early breeding season in establishment probability. However, our insights remain relevant to a broader range of animal species worldwide that share similar breeding behaviours and habitat preferences (Bronson 2009; Jones 2001; Lack 1950; Pankhurst and Porter 2003; Stouffer, Johnson, and Bierregaard 2013; Wells 2007).

## 6 Conclusion

An overwhelming amount of evidence postulates the positive relationship between propagule pressure and establishment success: larger founding populations are much more likely to establish, assuming suitable environmental conditions (Cassey et al. 2018). But in reality, species colonisation, whether natural or human‐mediated, begins with small founding populations (Deredec and Courchamp 2007; Pili et al. 2023; Wilson et al. 2009). On this note, studies have consistently shown that the establishment rate of small founding populations is highly variable (Cassey et al. 2018; O'Reilly‐Nugent et al. 2016); and this variability has been widely rashly dismissed as due to context dependence, randomness, among other reasons (Catford et al. 2022; Caughley 1994; O'Reilly‐Nugent et al. 2016; Pickett and Cadenasso 1995). Because establishment is the most crucial step of the species colonisation process, the knowledge gap on what causal confounding factors explain the high variability in the establishment of small founding populations is a research oversight that undermines the foundation of theoretical and applied biogeography (Catford et al. 2022). We have shown here that establishment and, by extension, colonisation are complex ecological processes inadequately embodied in simple mathematical or correlative models, such as the propagule pressure null model. To produce more useful models for predicting population establishment, it is imperative to consider two essential sources of context dependence: landscape spatial heterogeneity and dynamic environmental conditions. Such an added layer of complexity is beneficial (Forrest and Miller‐Rushing 2010; O'Reilly‐Nugent et al. 2016; Pickett and Cadenasso 1995) in making generalisable insights on population establishment and species colonisation. More importantly, it is only through such complex models that robust, defensible, and transferable predictions of establishment and colonisation can be produced, which altogether are necessary for applied biogeography, such as in guiding reintroduction efforts and invasive alien species risk assessments and management (Buchadas et al. 2017; Catford et al. 2022; Cuddington et al. 2013; DeAngelis and Yurek 2016; Pili et al. 2022).

## Conflicts of Interest

The authors declare no conflicts of interest.

## Supporting information


**Appendix S1.** Modifications made to *virToad* to suite this study’s simulation experiments.

**Figure S1.** Supplementary results.

## Data Availability

The project's HexSim project folder (including the colonisation scenarios and the outputs of scenarios) can be accessed in FigShare at https://doi.org/10.26180/24265687. The R script for running the statistical analyses can be accessed in Zenodo at https://doi.org/10.5281/zenodo.13847798.

## References

[eva70027-bib-0001] Armstrong, D. P. , and H. U. Wittmer . 2011. “Incorporating Allee Effects Into Reintroduction Strategies.” Ecological Research 26: 687–695.

[eva70027-bib-0002] Blackburn, T. M. 2004. “Method in Macroecology.” Basic and Applied Ecology 5, no. 5: 401–412. 10.1016/j.baae.2004.08.002.

[eva70027-bib-0003] Blackburn, T. M. , P. Cassey , R. P. Duncan , K. L. Evans , and K. J. Gaston . 2004. “Avian Extinction and Mammalian Introductions on Oceanic Islands.” Science 305, no. 5692: 1955–1958. 10.1126/science.1101617. 15448269

[eva70027-bib-0004] Blackburn, T. M. , and B. McGill . 2019. “Macroecology and Invasion Biology.” Global Ecology and Biogeography 28, no. 1: 28–32. 10.1111/geb.12838.

[eva70027-bib-0005] Blackburn, T. M. , P. Pysek , S. Bacher , et al. 2011. “A Proposed Unified Framework for Biological Invasions.” Trends in Ecology & Evolution 26, no. 7: 333–339. 10.1016/j.tree.2011.03.023. 21601306

[eva70027-bib-0006] Brodie, S. , K. Yasumiba , M. Towsey , P. Roe , and L. Schwarzkopf . 2020. “Acoustic Monitoring Reveals Year‐Round Calling by Invasive Toads in Tropical Australia.” Bioacoustics 1–17: 125–141. 10.1080/09524622.2019.1705183.

[eva70027-bib-0007] Bronson, F. 2009. “Climate Change and Seasonal Reproduction in Mammals.” Philosophical Transactions of the Royal Society, B: Biological Sciences 364, no. 1534: 3331–3340. 10.1098/rstb.2009.0140 PMC2781850 19833645

[eva70027-bib-0008] Brown, G. P. , C. Kelehear , and R. Shine . 2011. “Effects of Seasonal Aridity on the Ecology and Behaviour of Invasive Cane Toads in the Australian Wet‐Dry Tropics.” Functional Ecology 25, no. 6: 1339–1347. 10.1111/j.1365-2435.2011.01888.x.

[eva70027-bib-0009] Brown, G. P. , B. L. Phillips , J. K. Webb , and R. Shine . 2006. “Toad on the Road: Use of Roads as Dispersal Corridors by Cane Toads (*Bufo marinus*) at an Invasion Front in Tropical Australia.” Biological Conservation 133, no. 1: 88–94. 10.1016/j.biocon.2006.05.020.

[eva70027-bib-0010] Brown, J. H. , and M. V. Lomolino . 1998. Biogeography. 2nd ed. United States MA: Sinauer Associates, Inc.

[eva70027-bib-0011] Buchadas, A. , A. S. Vaz , J. P. Honrado , et al. 2017. “Dynamic Models in Research and Management of Biological Invasions.” Journal of Environmental Management 196: 594–606. 28351824 10.1016/j.jenvman.2017.03.060

[eva70027-bib-0012] Calatayud, N. E. , M. Stoops , and B. S. Durrant . 2018. “Ovarian Control and Monitoring in Amphibians.” Theriogenology 109: 70–81. 29325879 10.1016/j.theriogenology.2017.12.005

[eva70027-bib-0013] Capinha, C. , F. Essl , H. Seebens , D. Moser , and H. M. Pereira . 2015. “The Dispersal of Alien Species Redefines Biogeography in the Anthropocene.” Science 348, no. 6240: 1248–1251. 26068851 10.1126/science.aaa8913

[eva70027-bib-0014] Carnell, R. 2022. “Lhs: Latin Hypercube Samples (Version 3.4.0): CRAN.” https://cran.rproject.org/web/packages/lhs/lhs.

[eva70027-bib-0015] Carpenter, C. C. , and J. C. Gillingham . 1987. “Water Hole Fidelity in the Marine Toad, *Bufo marinus* .” Journal of Herpetology 21, no. 2: 158–161.

[eva70027-bib-0016] Cassey, P. , S. Delean , J. L. Lockwood , J. S. Sadowski , and T. M. Blackburn . 2018. “Dissecting the Null Model for Biological Invasions: A Meta‐Analysis of the Propagule Pressure Effect.” PLoS Biology 16, no. 4: e2005987. 10.1371/journal.pbio.2005987. 29684017 PMC5933808

[eva70027-bib-0017] Cassey, P. , T. A. Prowse , and T. M. Blackburn . 2014. “A Population Model for Predicting the Successful Establishment of Introduced Bird Species.” Oecologia 175, no. 1: 417–428. 10.1007/s00442-014-2902-1. 24566638

[eva70027-bib-0018] Castro‐Diez, P. , A. S. Vaz , J. S. Silva , et al. 2019. “Global Effects of Non‐Native Tree Species on Multiple Ecosystem Services.” Biological Reviews of the Cambridge Philosophical Society 94, no. 4: 1477–1501. 10.1111/brv.12511. 30974048 PMC6850375

[eva70027-bib-0019] Catford, J. A. , J. R. U. Wilson , P. Pysek , P. E. Hulme , and R. P. Duncan . 2022. “Addressing Context Dependence in Ecology.” Trends in Ecology & Evolution 37, no. 2: 158–170. 10.1016/j.tree.2021.09.007. 34756764

[eva70027-bib-0020] Caughley, G. 1994. “Directions in Conservation Biology.” Journal of Animal Ecology 63: 215–244.

[eva70027-bib-0021] Chambers, J. M. , A. E. Freeny , and R. M. Heiberger . 2017. “Analysis of Variance: Designed Experiments.” In Statistical Models in S, edited by J. M. Chambers and T. J. Hastie, 145–193. London, UK: Routledge.

[eva70027-bib-0022] Chapple, D. G. , S. M. Simmonds , and B. B. Wong . 2012. “Can Behavioral and Personality Traits Influence the Success of Unintentional Species Introductions?” Trends in Ecology & Evolution 27, no. 1: 57–64. 10.1016/j.tree.2011.09.010. 22001529

[eva70027-bib-0023] Child, T. , B. L. Phillips , and R. Shine . 2008. “Abiotic and Biotic Influences on the Dispersal Behavior of Metamorph Cane Toads (*Bufo marinus*) in Tropical Australia.” Journal of Experimental Zoology Part A: Ecological Genetics and Physiology 309, no. 4: 215–224. 10.1002/jez.450. 18288694

[eva70027-bib-0024] Child, T. , B. L. Phillips , and R. Shine . 2009. “Does Desiccation Risk Drive the Distribution of Juvenile Cane Toads (*Bufo marinus*) in Tropical Australia?” Journal of Tropical Ecology 25, no. 2: 193–200. 10.1017/s0266467408005695.

[eva70027-bib-0025] Clarke, G. S. , R. Shine , and B. L. Phillips . 2019. “Whispers on the Wind: Male Cane Toads Modify Mate Searching and Amplexus Tactics Based on Calls From Other Males.” Animal Behaviour 153: 131–136. 10.1016/j.anbehav.2019.05.008.

[eva70027-bib-0026] Cohen, M. P. 1995. Ecology of Two Populations of Bufo marinus in North‐Eastern Australia. (Doctor of Philosophy). Townsville, QLD, Australia: James Cook University.

[eva70027-bib-0027] Colautti, R. I. , I. A. Grigorovich , and H. J. MacIsaac . 2006. “Propagule Pressure: A Null Model for Biological Invasions.” Biological Invasions 8, no. 5: 1023–1037. 10.1007/s10530-005-3735-y.

[eva70027-bib-0028] Courchamp, F. , T. Clutton‐Brock , and B. Grenfell . 1999. “Inverse Density Dependence and the Allee Effect.” Trends in Ecology & Evolution 14, no. 10: 405–410. 10481205 10.1016/s0169-5347(99)01683-3

[eva70027-bib-0029] Crossland, M. R. , M. N. Hearnden , L. Pizzatto , R. A. Alford , and R. Shine . 2011. “Why Be a Cannibal? The Benefits to Cane Toad, *Rhinella marina* [=*Bufo marinus*], Tadpoles of Consuming Conspecific Eggs.” Animal Behaviour 82, no. 4: 775–782. 10.1016/j.anbehav.2011.07.009.

[eva70027-bib-0030] Cuddington, K. , M.‐J. Fortin , L. Gerber , et al. 2013. “Process‐Based Models Are Required to Manage Ecological Systems in a Changing World.” Ecosphere 4, no. 2: 1–12.

[eva70027-bib-0031] Cumming, G. 2009. “Inference by Eye: Reading the Overlap of Independent Confidence Intervals.” Statistics in Medicine 28, no. 2: 205–220. 10.1002/sim.3471. 18991332

[eva70027-bib-0032] Davis, M. A. , and K. Thompson . 2000. “Eight Ways to Be a Colonizer; Two Ways to Be an Invader: A Proposed Nomenclature Scheme for Invasion Ecology.” Bulletin of the Ecological Society of America 81, no. 3: 226–230.

[eva70027-bib-0033] DeAngelis, D. L. , and S. Yurek . 2016. “Spatially Explicit Modeling in Ecology: A Review.” Ecosystems 20, no. 2: 284–300. 10.1007/s10021-016-0066-z.

[eva70027-bib-0034] Deredec, A. , and F. Courchamp . 2007. “Importance of the Allee Effect for Reintroductions.” Ecoscience 14, no. 4: 440–451.

[eva70027-bib-0035] Devictor, V. , J. Clavel , R. Julliard , et al. 2010. “Defining and Measuring Ecological Specialization.” Journal of Applied Ecology 47, no. 1: 15–25. 10.1111/j.1365-2664.2009.01744.x.

[eva70027-bib-0036] Drake, J. A. , F. C. Dicastri , R. Groves , et al. 1989. Biological Invasins a Global Perspective. Chichester, NYC: Scope 37.

[eva70027-bib-0037] Duncan, R. P. , T. M. Blackburn , S. Rossinelli , and S. Bacher . 2014. “Quantifying Invasion Risk: The Relationship Between Establishment Probability and Founding Population Size.” Methods in Ecology and Evolution 5, no. 11: 1255–1263.

[eva70027-bib-0038] Elliott‐Graves, A. 2015. “The Problem of Prediction in Invasion Biology.” Biology & Philosophy 31, no. 3: 373–393. 10.1007/s10539-015-9504-0.

[eva70027-bib-0039] Forrest, J. , and A. J. Miller‐Rushing . 2010. “Toward a Synthetic Understanding of the Role of Phenology in Ecology and Evolution.” Philosophical Transactions of the Royal Society B: Biological Sciences 365: 3101–3112. 10.1098/rstb.2010.0145. PMC2981948 20819806

[eva70027-bib-0040] Forsyth, D. M. , R. P. Duncan , M. Bomford , and G. Moore . 2004. “Climatic Suitability, Life‐History Traits, Introduction Effort, and the Establishment and Spread of Introduced Mammals in Australia.” Conservation Biology 18, no. 2: 557–569.

[eva70027-bib-0041] Frederiksen, M. , M. P. Harris , F. Daunt , P. Rothery , and S. Wanless . 2004. “Scale‐Dependent Climate Signals Drive Breeding Phenology of Three Seabird Species.” Global Change Biology 10, no. 7: 1214–1221.

[eva70027-bib-0042] Gascoigne, J. , L. Berec , S. Gregory , and F. Courchamp . 2009. “Dangerously Few Liaisons: A Review of Mate‐Finding Allee Effects.” Population Ecology 51: 355–372.

[eva70027-bib-0043] Gaston, K. J. , A. G. Jones , C. Hanel , and S. L. Chown . 2003. “Rates of Species Introduction to a Remote Oceanic Island.” Proceedings of the Biological Sciences 270, no. 1519: 1091–1098. 10.1098/rspb.2003.2332. PMC1691340 12803900

[eva70027-bib-0044] Gelman, A. , and J. Hill . 2006. Data Analysis Using Regression and Multilevel/Hierarchical Models. New York: Cambridge University Press.

[eva70027-bib-0045] Gelman, A. , Y.‐S. Su , M. Yajima , et al. 2013. Package ‘Arm’: Data Analysis Using Regression and Multilevel/Hierarchical Models. Cambridge, UK: Cambridge University Press.

[eva70027-bib-0046] Gleditsch, J. M. , J. E. Behm , and M. R. Helmus . 2023. “The Great Acceleration of Island Saturation by Species Introductions in the Anthropocene Has Altered Species‐Area Relationships.” bioRxiv: 523426. 10.1101/2023.01.10.523426.

[eva70027-bib-0047] González‐Bernal, E. , G. P. Brown , M. S. Crowther , and R. Shine . 2015. “Sex and Age Differences in Habitat Use by Invasive Cane Toads (*Rhinella marina*) and a Native Anuran (*Cyclorana australis*) in the Australian Wet‐Dry Tropics.” Austral Ecology 40, no. 8: 953–961. 10.1111/aec.12279.

[eva70027-bib-0048] Gonzalez‐Bernal, E. , M. Greenlees , G. P. Brown , and R. Shine . 2012. “Cane Toads on Cowpats: Commercial Livestock Production Facilitates Toad Invasion in Tropical Australia.” PLoS One 7, no. 11: e49351. 10.1371/journal.pone.0049351. 23145158 PMC3492292

[eva70027-bib-0049] González‐Bernal, E. , M. J. Greenlees , G. P. Brown , and R. Shine . 2016. “Toads in the Backyard: Why Do Invasive Cane Toads (*Rhinella marina*) Prefer Buildings to Bushland?” Population Ecology 58, no. 2: 293–302. 10.1007/s10144-016-0539-0.

[eva70027-bib-0050] Hayes, K. R. , and S. C. Barry . 2007. “Are There any Consistent Predictors of Invasion Success?” Biological Invasions 10, no. 4: 483–506. 10.1007/s10530-007-9146-5.

[eva70027-bib-0051] Hearnden, M. N. 1991. The Reproductive and Larval Ecology of Bufo marinus (Anura: Bufonidae) (Doctor of Philosophy). Townsville, QLD, Australia: James Cook University.

[eva70027-bib-0052] Hoffmann, B. D. , and F. Courchamp . 2016. “Biological Invasions and Natural Colonisations: Are They That Different?” NeoBiota 29: 1–14. 10.3897/neobiota.29.6959.

[eva70027-bib-0053] Hosmer, D. W., Jr. , S. Lemeshow , and R. X. Sturdivant . 2013. Applied Logistic Regression. Vol. 398. Hoboken, NJ, USA: John Wiley & Sons.

[eva70027-bib-0054] Jeschke, J. M. , F. Keesing , and R. S. Ostfeld . 2013. “Novel Organisms: Comparing Invasive Species, GMOs, and Emerging Pathogens.” Ambio 42, no. 5: 541–548. 10.1007/s13280-013-0387-5. 23456779 PMC3698323

[eva70027-bib-0055] Joly, P. 2019. “Behavior in a Changing Landscape: Using Movement Ecology to Inform the Conservation of Pond‐Breeding Amphibians.” Frontiers in Ecology and Evolution 7: 155. 10.3389/fevo.2019.00155.

[eva70027-bib-0056] Jones, J. 2001. “Habitat Selection Studies in Avian Ecology: A Critical Review.” Auk 118, no. 2: 557–562.

[eva70027-bib-0057] Jørgensen, C. B. 1991. “Water Economy in the Life of a Terrestrial Anuran, the Toad *Bufo bufo* .” Kongelige Danske Videnskabernes Selskab 39: 1–30.

[eva70027-bib-0058] Kelehear, C. , and R. Shine . 2020. “Tradeoffs Between Dispersal and Reproduction at an Invasion Front of Cane Toads in Tropical Australia.” Scientific Reports 10, no. 1: 486. 10.1038/s41598-019-57391-x. 31949254 PMC6965623

[eva70027-bib-0059] Kolar, C. S. , and D. M. Lodge . 2002. “Ecological Predictions and Risk Assessment for Alien Fishes in North America.” Science 298, no. 5596: 1233–1236. 10.1126/science.1075753. 12424378

[eva70027-bib-0060] Lack, D. 1950. “The Breeding Seasons of European Birds.” IBIS 92, no. 2: 288–316.

[eva70027-bib-0061] Leung, B. , N. Roura‐Pascual , S. Bacher , et al. 2012. “TEASIng Apart Alien Species Risk Assessments: A Framework for Best Practices.” Ecology Letters 15, no. 12: 1475–1493. 10.1111/ele.12003. 23020170

[eva70027-bib-0062] Lockwood, J. L. , P. Cassey , and T. Blackburn . 2005. “The Role of Propagule Pressure in Explaining Species Invasions.” Trends in Ecology & Evolution 20, no. 5: 223–228. 10.1016/j.tree.2005.02.004. 16701373

[eva70027-bib-0063] MacArthur, R. H. , and E. O. Wilson . 2001. The Theory of Island Biogeography. Vol. 1. Princeton, NJ, USA: Princeton University Press.

[eva70027-bib-0064] March‐Salas, M. , and L. R. Pertierra . 2020. “Warmer and Less Variable Temperatures Favour an Accelerated Plant Phenology of Two Invasive Weeds Across Sub‐Antarctic Macquarie Island.” Austral Ecology 45, no. 5: 572–585. 10.1111/aec.12872.

[eva70027-bib-0065] Martin, R. O. , L. Sebele , A. Koeslag , O. Curtis , F. Abadi , and A. Amar . 2014. “Phenological Shifts Assist Colonisation of a Novel Environment in a Range‐Expanding Raptor.” Oikos 123, no. 12: 1457–1468.

[eva70027-bib-0066] McCann, S. , M. Crossland , M. Greenlees , and R. Shine . 2020. “Field Trials of Chemical Suppression of Embryonic Cane Toads (*Rhinella marina*) by Older Conspecifics.” Ecology and Evolution 10, no. 18: 10177–10185. 10.1002/ece3.6678. 33005373 PMC7520185

[eva70027-bib-0067] McGeoch, M. A. , P. Genovesi , P. J. Bellingham , M. J. Costello , C. McGrannachan , and A. Sheppard . 2015. “Prioritizing Species, Pathways, and Sites to Achieve Conservation Targets for Biological Invasion.” Biological Invasions 18, no. 2: 299–314. 10.1007/s10530-015-1013-1.

[eva70027-bib-0068] McKay, M. D. , R. J. Beckman , and W. J. Conover . 2000. “A Comparison of Three Methods for Selecting Values of Input Variables in the Analysis of Output From a Computer Code.” Technometrics 42, no. 1: 55–61.

[eva70027-bib-0069] Melbourne, B. A. , and A. Hastings . 2009. “Highly Variable Spread Rates in Replicated Biological Invasions: Fundamental Limits to Predictability.” Science 325, no. 5947: 1536–1539. 19762641 10.1126/science.1176138

[eva70027-bib-0070] Molles, L. , A. Calcott , D. Peters , et al. 2008. “Acoustic Anchoring and the Successful Translocation of North Island Kokako (Callaeas Cinerea Wilsoni) to a New Zealand Mainland Management Site Within Continuous Forest.” Notorni 55: 57–68.

[eva70027-bib-0071] Muller, B. J. , and L. Schwarzkopf . 2017. “Relative Effectiveness of Trapping and Hand‐Capture for Controlling Invasive Cane Toads (*Rhinella marina*).” International Journal of Pest Management 64, no. 2: 185–192. 10.1080/09670874.2017.1363443.

[eva70027-bib-0072] Olden, J. D. , N. Leroy Poff , M. R. Douglas , M. E. Douglas , and K. D. Fausch . 2004. “Ecological and Evolutionary Consequences of Biotic Homogenization.” Trends in Ecology & Evolution 19, no. 1: 18–24. 10.1016/j.tree.2003.09.010. 16701221

[eva70027-bib-0073] O'Reilly‐Nugent, A. , R. Palit , A. Lopez‐Aldana , M. Medina‐Romero , E. Wandrag , and R. P. Duncan . 2016. “Landscape Effects on the Spread of Invasive Species.” Current Landscape Ecology Reports 1: 107–114.

[eva70027-bib-0074] Pankhurst, N. , and M. Porter . 2003. “Cold and Dark or Warm and Light: Variations on the Theme of Environmental Control of Reproduction.” Fish Physiology and Biochemistry 28: 385–389.

[eva70027-bib-0075] Phillips, B. L. , G. P. Brown , M. Greenlees , J. K. Webb , and R. Shine . 2007. “Rapid Expansion of the Cane Toad (*Bufo marinus*) Invasion Front in Tropical Australia.” Austral Ecology 32, no. 2: 169–176.

[eva70027-bib-0076] Pickett, S. T. , and M. L. Cadenasso . 1995. “Landscape Ecology: Spatial Heterogeneity in Ecological Systems.” Science 269, no. 5222: 331–334. 17841249 10.1126/science.269.5222.331

[eva70027-bib-0077] Pili, A. N. , R. Tingley , D. G. Chapple , and N. H. Schumaker . 2022. “virToad: Simulating the Spatiotemporal Population Dynamics and Management of a Global Invader.” Landscape Ecology 37, no. 9: 2273–2292.

[eva70027-bib-0078] Pili, A. N. , R. Tingley , D. van Winkel , L. Maria , and D. G. Chapple . 2023. “The Escalating Global Problem of Accidental Human‐Mediated Transport of Alien Species: A Case Study Using Alien Herpetofauna Interceptions in New Zealand.” Biological Conservation 278: 109860.

[eva70027-bib-0079] Pysek, P. , P. E. Hulme , D. Simberloff , et al. 2020. “Scientists' Warning on Invasive Alien Species.” Biological Reviews of the Cambridge Philosophical Society 95: 1511–1534. 10.1111/brv.12627. 32588508 PMC7687187

[eva70027-bib-0080] R Core Team . 2023. R: A Language and Environment for Statistical Computing (Version 4.3.1). Vienna, Austria: R Foundation for Statistical Computing. https://cran.r‐project.org.

[eva70027-bib-0081] Rago, A. , G. M. While , and T. Uller . 2012. “Introduction Pathway and Climate Trump Ecology and Life History as Predictors of Establishment Success in Alien Frogs and Toads.” Ecology and Evolution 2, no. 7: 1437–1445. 10.1002/ece3.261. 22957152 PMC3434934

[eva70027-bib-0082] Richardson, D. M. , P. Pyšek , M. Rejmánek , M. G. Barbour , F. D. Panetta , and C. J. West . 2000. “Naturalization and Invasion of Alien Plants: Concepts and Definitions.” Diversity and Distributions 6, no. 2: 93–107.

[eva70027-bib-0083] Roura‐Pascual, N. , C. Hui , T. Ikeda , et al. 2011. “Relative Roles of Climatic Suitability and Anthropogenic Influence in Determining the Pattern of Spread in a Global Invader.” Proceedings of the National Academy of Sciences of the United States of America 108, no. 1: 220–225. 10.1073/pnas.1011723108. 21173219 PMC3017164

[eva70027-bib-0084] Schumaker, N. H. , and A. Brookes . 2018. “HexSim: A Modeling Environment for Ecology and Conservation.” Landscape Ecology 33: 197–211. 10.1007/s10980-017-0605-9. 29545713 PMC5846496

[eva70027-bib-0085] Schwarzkopf, L. , and R. Alford . 1996. “Desiccation and Shelter‐Site Use in a Tropical Amphibian: Comparing Toads With Physical Models.” Functional Ecology 10, no. 2: 193–200.

[eva70027-bib-0086] Seebacher, F. , and R. A. Alford . 2002. “Shelter Microhabitats Determine Body Temperature and Dehydration Rates of a Terrestrial Amphibian (*Bufo marinus*).” Journal of Herpetology 36: 69–75.

[eva70027-bib-0087] Seebens, H. , T. M. Blackburn , E. E. Dyer , et al. 2017. “No Saturation in the Accumulation of Alien Species Worldwide.” Nature Communications 8: 14435. 10.1038/ncomms14435. PMC5316856 28198420

[eva70027-bib-0088] Seebens, H. , T. M. Blackburn , E. E. Dyer , et al. 2018. “Global Rise in Emerging Alien Species Results From Increased Accessibility of New Source Pools.” Proceedings of the National Academy of Sciences of the United States of America 115, no. 10: E2264–E2273. 10.1073/pnas.1719429115. 29432147 PMC5877962

[eva70027-bib-0089] Segrestin, J. , M. Bernard‐Verdier , C. Violle , J. Richarte , M. L. Navas , and E. Garnier . 2018. “When Is the Best Time to Flower and Disperse? A Comparative Analysis of Plant Reproductive Phenology in the Mediterranean.” Functional Ecology 32, no. 7: 1770–1783.

[eva70027-bib-0090] Sentis, A. , J. M. Montoya , and M. Lurgi . 2021. “Warming Indirectly Increases Invasion Success in Food Webs.” Proceedings of the Biological Sciences 288, no. 1947: 20202622. 10.1098/rspb.2020.2622. PMC8059653 33726601

[eva70027-bib-0091] Simberloff, D. 2010. “Charles Elton: Neither Founder Nor Siren, but Prophet.” In Fifty Years of Invasion Ecology: The Legacy of Charles Elton, edited by D. M. Richardson , 11–24. Hoboken, NJ, USA: Blackwell Publishing Ltd. .

[eva70027-bib-0092] Smart, A. S. , R. Tingley , and B. L. Phillips . 2020. “Estimating the Benefit of Quarantine: Eradicating Invasive Cane Toads From Islands.” NeoBiota 60: 117–136. 10.3897/neobiota.60.34941.

[eva70027-bib-0093] Soberon, J. 2007. “Grinnellian and Eltonian Niches and Geographic Distributions of Species.” Ecology Letters 10, no. 12: 1115–1123. 10.1111/j.1461-0248.2007.01107.x. 17850335

[eva70027-bib-0094] Spring, D. , and T. Kompas . 2017. Groote Eylandt Mining Company Operation (GEMCO) Cane Toad Management Review. Northern Territory, Australia: Groote Eylandt Mining Company Pty Ltd.

[eva70027-bib-0095] Stein, M. 1987. “Large Sample Properties of Simulations Using Latin Hypercube Sampling.” Technometrics 29, no. 2: 143–151.

[eva70027-bib-0096] Stephens, P. A. , and W. J. Sutherland . 1999. “Consequences of the Allee Effect for Behaviour, Ecology and Conservation.” Trends in Ecology & Evolution 14, no. 10: 401–405. 10481204 10.1016/s0169-5347(99)01684-5

[eva70027-bib-0097] Stigall, A. L. 2019. “The Invasion Hierarchy: Ecological and Evolutionary Consequences of Invasions in the Fossil Record.” Annual Review of Ecology, Evolution, and Systematics 50, no. 1: 355–380. 10.1146/annurev-ecolsys-110617-062638.

[eva70027-bib-0098] Stone, M. 1974. “Cross‐Validatory Choice and Assessment of Statistical Predictions.” Journal of the Royal Statistical Society: Series B: Methodological 36, no. 2: 111–133.

[eva70027-bib-0099] Stouffer, P. C. , E. I. Johnson , and R. O. Bierregaard Jr. 2013. “Breeding Seasonality in Central Amazonian Rainforest Birds.” Auk 130, no. 3: 529–540.

[eva70027-bib-0100] Stringham, O. C. , and J. L. Lockwood . 2021. “Managing Propagule Pressure to Prevent Invasive Species Establishments: Propagule Size, Number, and Risk–Release Curve.” Ecological Applications 31, no. 4: e02314. 33636036 10.1002/eap.2314

[eva70027-bib-0101] Swets, J. A. 1988. “Measuring the Accuracy of Diagnostic Systems.” Science 240, no. 4857: 1285–1293. 10.1126/science.3287615. 3287615

[eva70027-bib-0102] Tukey, J. W. 1949. “Comparing Individual Means in the Analysis of Variance.” Biometrics 5: 99–114. 18151955

[eva70027-bib-0103] Vila, M. , J. L. Espinar , M. Hejda , et al. 2011. “Ecological Impacts of Invasive Alien Plants: A Meta‐Analysis of Their Effects on Species, Communities and Ecosystems.” Ecology Letters 14, no. 7: 702–708. 10.1111/j.1461-0248.2011.01628.x. 21592274

[eva70027-bib-0104] Vilà, M. , and P. E. Hulme . 2017. Impact of Biological Invasions on Ecosystem Services. Vol. 12. Cham, Switzerland: Springer.

[eva70027-bib-0105] Visser, M. E. , and C. Both . 2005. “Shifts in Phenology Due to Global Climate Change: The Need for a Yardstick.” Proceedings of the Royal Society B: Biological Sciences 272, no. 1581: 2561–2569. 10.1098/rspb.2005.3356 PMC1559974 16321776

[eva70027-bib-0106] Wainright, C. A. , C. C. Muhlfeld , J. J. Elser , S. L. Bourret , and S. P. Devlin . 2021. “Species Invasion Progressively Disrupts the Trophic Structure of Native Food Webs.” Proceedings of the National Academy of Sciences of the United States of America 118, no. 45: e2102179118. 10.1073/pnas.2102179118. 34725150 PMC8609295

[eva70027-bib-0107] Walter, J. A. , D. M. Johnson , and K. J. Haynes . 2017. “Spatial Variation in Allee Effects Influences Patterns of Range Expansion.” Ecography 40, no. 1: 179–188.

[eva70027-bib-0108] Ward‐Fear, G. , M. J. Greenlees , and R. Shine . 2016. “Toads on Lava: Spatial Ecology and Habitat Use of Invasive Cane Toads (*Rhinella marina*) in Hawai'i.” PLoS One 11, no. 3: e0151700. 10.1371/journal.pone.0151700. 27027738 PMC4814139

[eva70027-bib-0109] Wells, K. D. 2007. The Ecology and Behavior of Amphibians. 1st ed. Chicago, IL, USA: University of Chicago Press.

[eva70027-bib-0110] White, A. W. , and R. Shine . 2009. “The Extra‐Limital Spread of an Invasive Species via ‘Stowaway’ Dispersal: Toad to Nowhere?” Animal Conservation 12, no. 1: 38–45. 10.1111/j.1469-1795.2008.00218.x.

[eva70027-bib-0111] Williamson, M. , H. L. Kornberg , M. Holdgate , A. Gray , and G. R. Conway . 1986. Preface‐The British Contribution to the SCOPE Programme on the Ecology of Biological Invasions. London: Royal Society.

[eva70027-bib-0112] Wilson, E. O. , and R. H. MacArthur . 1967. The Theory of Island Biogeography. Princeton, NJ, USA: Princeton University Press.

[eva70027-bib-0113] Wilson, J. R. , E. E. Dormontt , P. J. Prentis , A. J. Lowe , and D. M. Richardson . 2009. “Something in the Way You Move: Dispersal Pathways Affect Invasion Success.” Trends in Ecology & Evolution 24, no. 3: 136–144. 10.1016/j.tree.2008.10.007. 19178981

[eva70027-bib-0114] Yasumiba, K. , R. A. Alford , and L. Schwarzkopf . 2015. “Why Do Male and Female Cane Toads, *Rhinella marina*, Respond Differently to Advertisement Calls?” Animal Behaviour 109: 141–147. 10.1016/j.anbehav.2015.08.015.

[eva70027-bib-0115] Yasumiba, K. , R. A. Alford , and L. Schwarzkopf . 2016. “Seasonal Reproductive Cycles of Cane Toads and Their Implications for Control.” Herpetologica 72, no. 4: 288–292. 10.1655/Herpetologica-D-15-00048.1.

