# Supplementary material for: Landscape Heterogeneity and Environmental Dynamics Improve Predictions of Establishment Success of Colonising Small Founding Populations
Source: Evol Appl. 2024 Oct 21;17(10):e70027. doi: 10.1111/eva.70027 (PMC11493551; doi:10.1111/eva.70027)
Supplement: Supplementary file 1 — Appendix S1. Modifications made to virToad to suite this study’s simulation experiments. [file EVA-17-e70027-s002.docx]

**Supplementary Information S1**

Pili, A. N., Schumaker, N. H., Camacho-Cervantes, M., Tingley, R., and Chapple, D. G. (2024). Landscape heterogeneity and environmental dynamics improve predictions of establishment success of colonising small founding populations. Evolutionary Applications.

The following modifications in *virToad* were made:

- As in the case in the wild, upon release from a transport vehicle (i.e., introduction), simulated toads become temporarily more active and dispersive. This is a stress response to transportation, translocation, and encountering new environments (Pettit et al. 2016, Pettit et al. 2017, Pettit et al. 2020). For five days from introduction, simulated toads are temporarily more active and dispersive; that is, for 5 days, their probability of being active and dispersal distance is equivalent to a cane toad in the invasion front.
- Simulated cane toads are repulsed from dispersing into hexagon cells located in the sea.
- Ephemeral streams have water only during the wet season. And they are dry during the dry season.
- During the wet season, ephemeral streams have non-flowing small waterbodies along its shoreline that are conducive for reproduction and development (1% randomly selected among hexagons on shoreline of ephemeral streams).
- Small waterbodies that provide water for rehydration, and are conducive for reproduction and development, are found along road drainages, human inhabited areas, and economic areas year-long. This was randomly assigned (1%) among hexagons on roads, human habitation, and economic zones.
