# Supplementary material for: Landscape Heterogeneity and Environmental Dynamics Improve Predictions of Establishment Success of Colonising Small Founding Populations
Source: Evol Appl. 2024 Oct 21;17(10):e70027. doi: 10.1111/eva.70027 (PMC11493551; doi:10.1111/eva.70027)
Supplement: Supplementary file 2 — Figure S1. Supplementary results. [file EVA-17-e70027-s001.docx]

**Supplementary Information S2**

Pili, A. N., Schumaker, N. H., Camacho-Cervantes, M., Tingley, R., and Chapple, D. G. (2024). Landscape heterogeneity and environmental dynamics improve predictions of establishment success of colonising small founding populations. Evolutionary Applications.


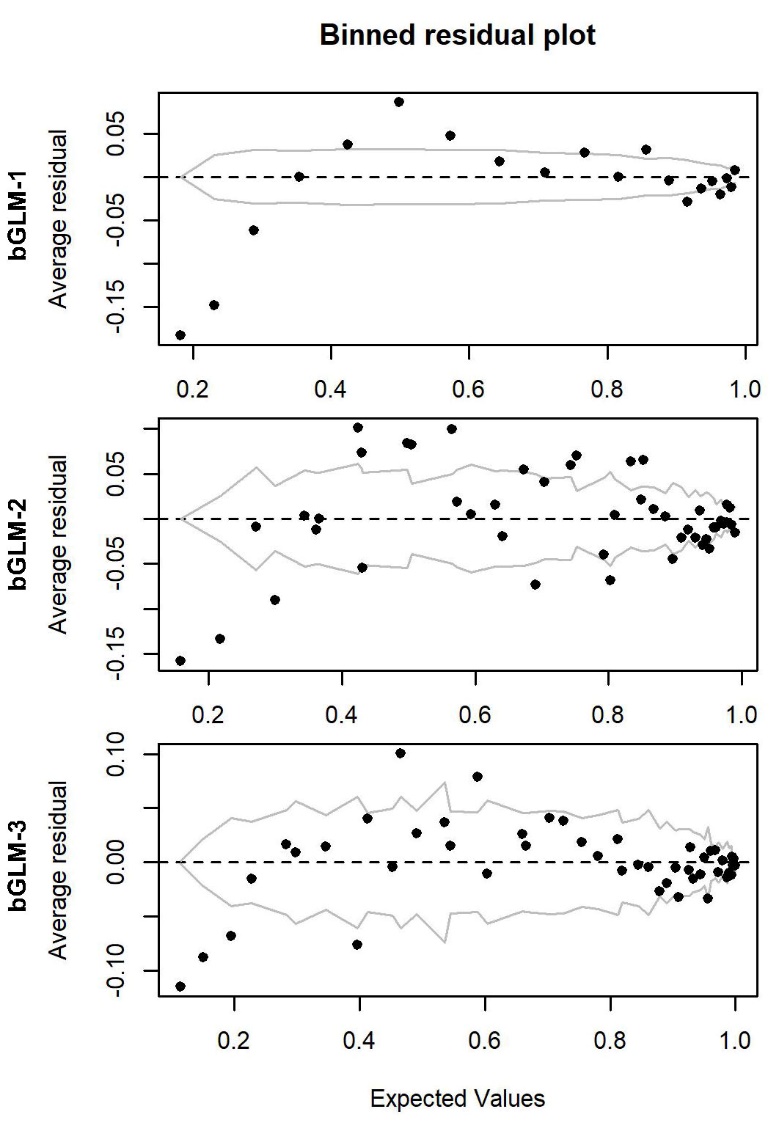


**Figure S2.1.** Binned residual plots of static bGLM-1 (top), bGLM-2 (middle), bGLM-3 (bottom). Here, predictions are first divided into 50 bins based on their fitted values. Then, the average residual versus the average fitted value for each bin is then plotted. The grey lines represent the lower and upper bands of 95% confidence interval. Points that fall outside the condifence interval bands indicate overprediction (negative values) or underprediciton (positive values). Among all models, there are more outliers among the residuals than we would expect from chance alone.


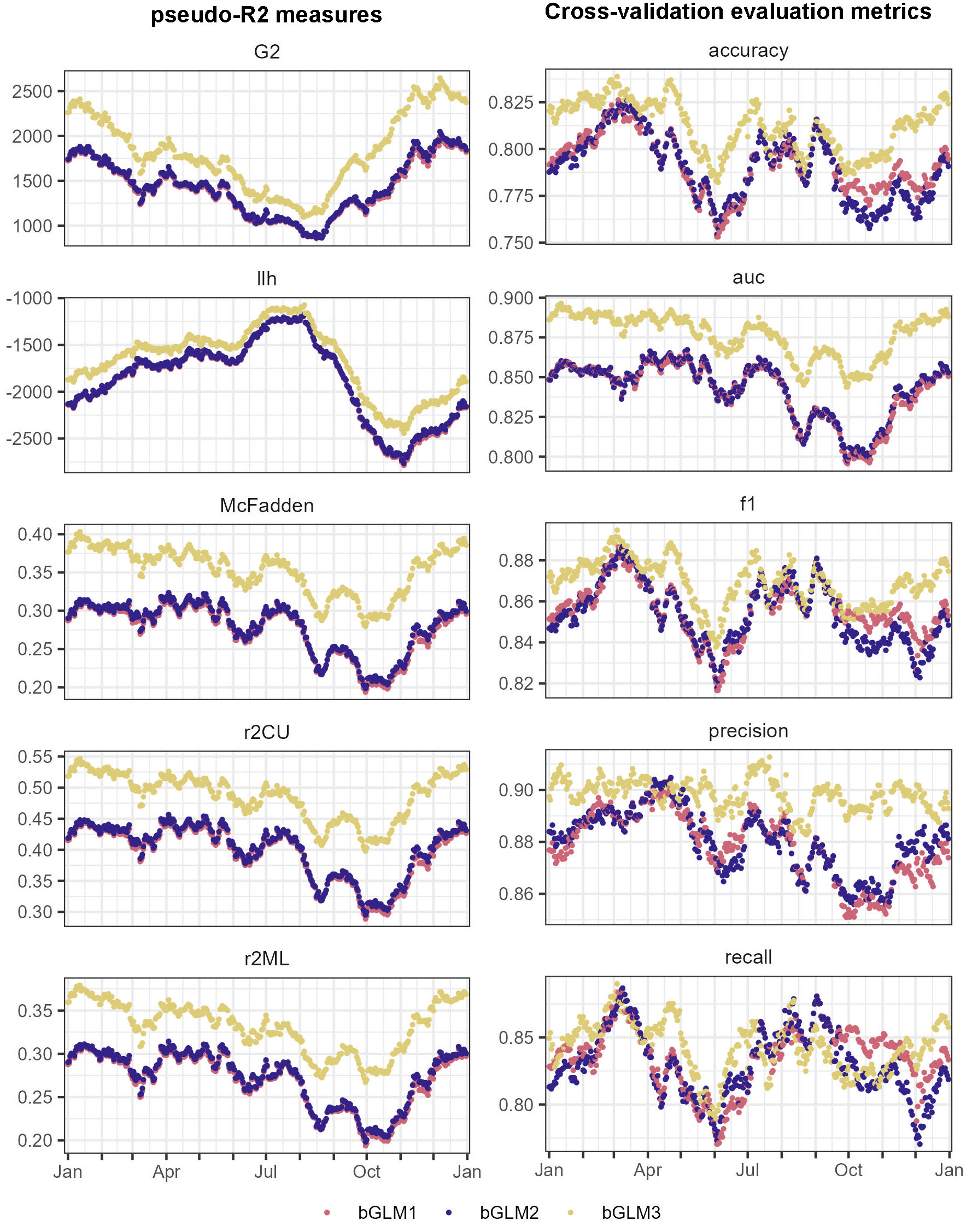


Figure S2.2. Variability of model goodness-of-fit over time, based on dynamic binomial GLMs.

**
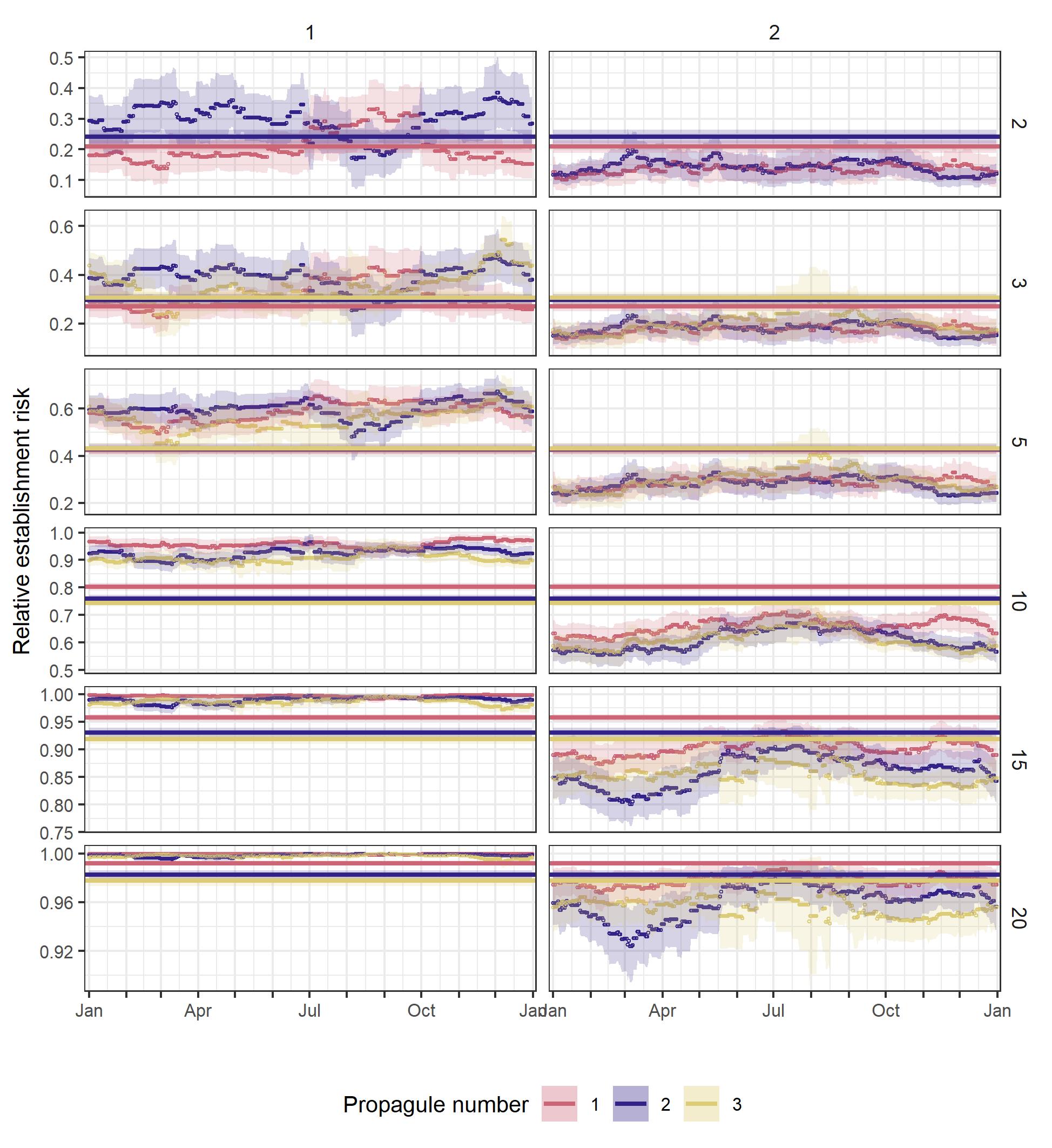
**

**Figure S2.3.** Comparison of the predicted establishment probability of dynamic-bGLM-3 and bGLM-2 (colored line). Ribbons indicate the 95% Confidence Intervals of predictions.

**
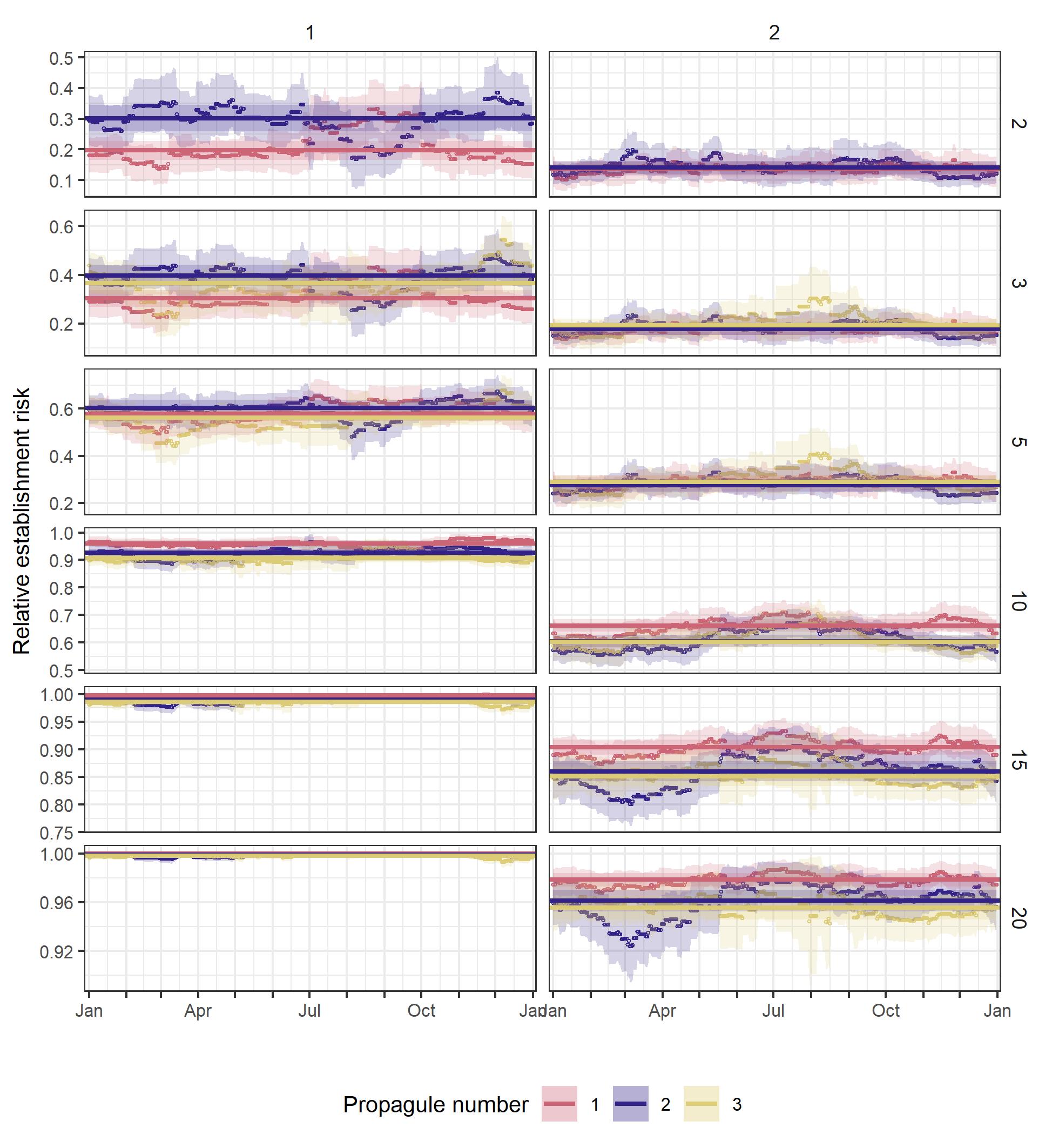
**

**Figure S2.4.** Comparison of the predicted establishment probability of r dynamic bGLM-3 and bGLM-3 (colored line). Ribbons indicate the 95% Confidence Intervals of predictions.
